# Yeast Bromodomain Factor 1 and Its Human Homolog TAF1 Play Conserved Roles in Promoting Homologous Recombination

**DOI:** 10.1002/advs.202100753

**Published:** 2021-05-30

**Authors:** Haoyang Peng, Simin Zhang, Yihan Peng, Shuangyi Zhu, Xin Zhao, Xiaocong Zhao, Shuangshuang Yang, Guangxue Liu, Yang Dong, Xiaoli Gan, Qing Li, Xinghua Zhang, Huadong Pei, Xuefeng Chen

**Affiliations:** ^1^ Hubei Key Laboratory of Cell Homeostasis College of Life Sciences and the Institute for Advanced Studies Wuhan University Wuhan 430072 China; ^2^ Department of Biochemistry and Molecular Medicine George Washington University School of Medicine and Health Science Washington DC 20037 USA; ^3^ State Key Laboratory of Protein and Plant Gene Research School of Life Sciences and Peking‐Tsinghua Center for Life Sciences Peking University Beijing 100871 China

**Keywords:** Bromodomain Factor 1 (Bdf1), histone acetylation, homologous recombination, Replication Protein A (RPA), TAF1

## Abstract

Histone acetylation is a key histone post‐translational modification that shapes chromatin structure, dynamics, and function. Bromodomain (BRD) proteins, the readers of acetyl‐lysines, are located in the center of the histone acetylation‐signaling network. How they regulate DNA repair and genome stability remains poorly understood. Here, a conserved function of the yeast Bromodomain Factor 1 (Bdf1) and its human counterpart TAF1 is reported in promoting DNA double‐stranded break repair by homologous recombination (HR). Depletion of either yeast *BDF1* or human TAF1, or disruption of their BRDs impairs DNA end resection, Replication Protein A (RPA) and Rad51 loading, and HR repair, causing genome instability and hypersensitivity to DNA damage. Mechanistically, it is shown that Bdf1 preferentially binds the DNA damage‐induced histone H4 acetylation (H4Ac) via the BRD motifs, leading to its chromatin recruitment. Meanwhile, Bdf1 physically interacts with RPA, and this interaction facilitates RPA loading in the chromatin context and the subsequent HR repair. Similarly, TAF1 also interacts with H4Ac or RPA. Thus, Bdf1 and TAF1 appear to share a conserved mechanism in linking the HR repair to chromatin acetylation in preserving genome integrity.

## Graphical abstract

How the bromodomain (BRD) proteins, readers of acetyl‐lysines, regulate DNA repair and genome stability remains partially understood. Here, a conserved function of the yeast Bromodomain Factor 1 (Bdf1) and its human counterpart TAF1 is shown in promoting DNA double‐stranded break repair by the homologous recombination pathway via physically facilitating Replication Protein A (RPA) loading on ssDNA with dispersed histones at break ends.

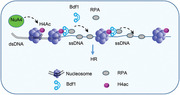

## 1 Introduction

Maintenance of genome stability is essential for cell homeostasis, the faithful transmission of genetic information into daughter cells, and the avoidance of cancer. DNA double‐stranded breaks (DSBs) are a highly deleterious form of DNA lesion threatening genome stability. To counteract the deleterious outcomes of DSBs, cells have evolved two highly conserved repair mechanisms, non‐homologous end joining (NHEJ) and homologous recombination (HR) pathways to repair DSBs.^[^ ^1^, ^2^ ^]^ Defects in DSB repair are related to various human diseases, including immune deficiencies, neurodegenerative disorders, and cancer.^[^ ^1^, ^2^ ^]^ HR is an essential mechanism for repairing DSBs from bacteria to human. It plays critical roles in the restart of collapsed replication forks and in meiosis.^[^ ^1^, ^2^ ^]^ HR utilizes a homologous template, usually a sister chromatid, to direct the repair and generally produces accurate repair products.^[^ ^1^, ^2^ ^]^ Deficiencies in HR cause genome instability and cancer.^[^ ^3^ ^]^

During HR, the 5’‐ends of DSBs are initially processed by the Mre11‐Rad50‐Xrs2 (MRX) complex (MRE11‐RAD50‐NBS1 in mammals) in conjunction with Sae2 (CtIP in mammals).^[^ ^4^ ^]^ Long‐range resection of the 5’‐ends is executed by the exonuclease Exo1 or the concerted actions of Sgs1 helicase (BLM or WRN in mammals) and Dna2 nuclease.^[^ ^5^, ^6^, ^7^, ^8^ ^]^ Replication Protein A (RPA), the conserved single‐stranded DNA (ssDNA) binding protein complex, binds the 3’‐tailed ssDNA revealed by resection to activate the DNA damage checkpoint and to initiate HR repair.^[^ ^1^, ^2^, ^9^, ^10^ ^]^ The recombinase Rad51 subsequently replaces RPA on ssDNA with the aid of Rad52 (BRCA2 in mammals), leading to the formation of the Rad51 nucleofilament that catalyzes homology search and strand invasion of homologous duplex DNA.^[^ ^2^ ^]^ The DNA synthesis from the 3’‐end of the invading strand extends D‐loop to complete the repair.^[^ ^2^ ^]^ The RPA complex lies in the core of the DNA damage response network. In addition to protecting ssDNA from nuclease attack or formation of DNA secondary structures, RPA also acts as a platform for recruiting and coordinating proteins involved in various DNA transactions. RPA is essential for DNA replication, repair, and recombination and plays indispensable roles in suppressing mutation and genome instability.^[^ ^11^, ^12^, ^13^, ^14^, ^15^ ^]^

In eukaryotes, all DNA transactions occur in the context of chromatin, so they are naturally affected by the versatile histone post‐translational modifications that shape the chromatin structures, dynamics, and functions. DSBs induce a number of different histone modifications on the damaged chromatin,^[^ ^16^ ^]^ suggesting a critical role of histone architecture in guiding DNA damage repair. One of the most important events is the acetylation of the N‐terminal tail of the core histone H4. Acetylation of histone H4 by NuA4 in yeast or TIP60 in mammals is essential for DSB repair and genome stability.^[^ ^17^, ^18^, ^19^ ^]^ However, how the histone acetylation signal is transduced to elicit DNA damage response and repair remains poorly understood.

Histone acetylation marks are written by histone acetyltransferase (HAT) and erased by histone deacetylase (HDAC). In eukaryotes, the conserved bromodomains (BRDs) function as the primary reader modules of acetyl‐lysines.^[^ ^20^, ^21^ ^]^ Thus, the BRD family proteins are placed at the center of the acetylation‐driven processes. Indeed, the BRD proteins have emerged as a fundamental mechanism by which histone acetylation acts *in trans* to regulate the DNA damage response and genome stability.^[^ ^22^, ^23^ ^]^ Yeast cells harbor over 10 BRD or BRD‐like proteins, while human cells have 46 BRD protein members.^[^ ^24^ ^]^ However, their precise functions in the DNA damage response and repair remain poorly understood. To fully understand how the histone acetylation‐BRD signaling network controls genome stability, it is essential to define the specific role for each of these BRD proteins.

In this study, we screened non‐essential yeast HATs, HDACs, and BRD proteins and found that the BRD family protein Bromodomain Factor 1 (Bdf1) and its human counterpart TAF1 play important roles in promoting DSB repair by HR and maintenance of genome stability. Bdf1 belongs to the BET subfamily member and harbors two consecutive BRDs and an extra terminal recruitment motif.^[^ ^25^ ^]^ Yeast Bdf1 corresponds to the C‐terminal half of human TAF1, a component of the TFIID complex.^[^ ^26^ ^]^ We found that both Bdf1 and TAF1 are important for DSB end resection, RPA and Rad51 loading, and HR repair. We showed that Bdf1/TAF1 binds acetylated histone H4 and RPA and that these interactions collectively facilitate RPA loading at DSBs and the repair by HR. Our studies provide novel insights into how histone acetylation and BRD proteins regulate DSB repair and how the ssDNA binding factor RPA assembles in the context of damaged chromatin.

## 2 Results

### 2.1 Screen for HATs, HDACs, and BRD Proteins Involved in DNA Damage Response

**Figure 1:**
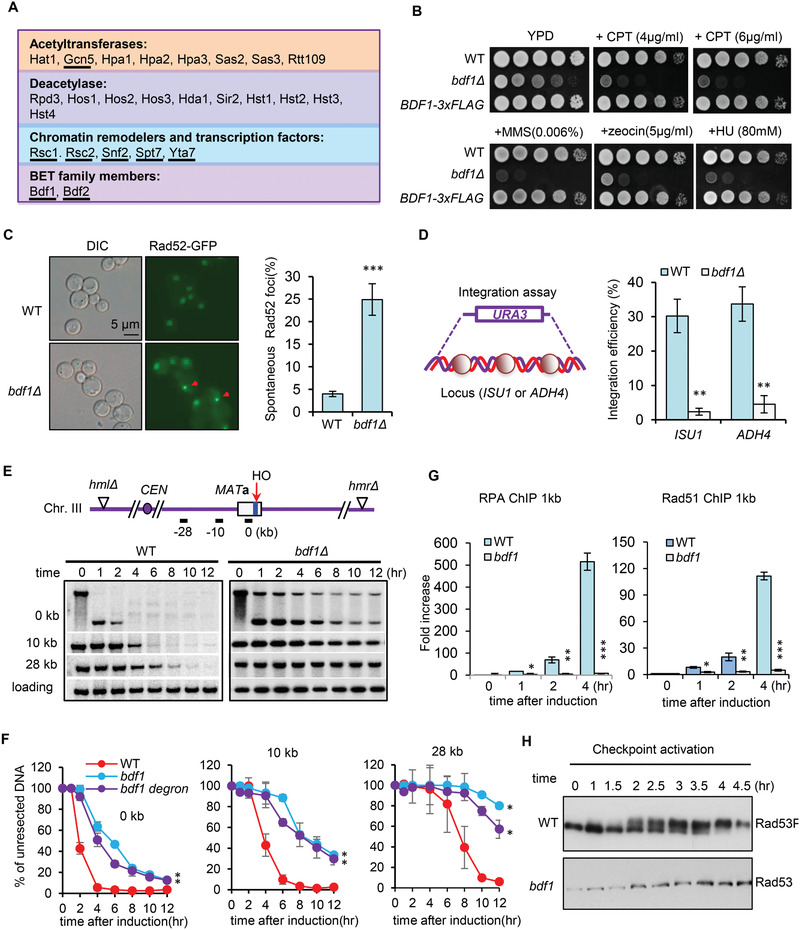
Bdf1 promotes DNA damage response and repair by HR. A) A list of yeast acetylation‐related mutants, including cells lacking acetyltransferases, deacetylases, or BRD‐containing proteins (underlined). B) DNA damage sensitivity test for indicated strains at indicated drug concentrations. C) Microscopy analysis and quantification of spontaneous Rad52‐YFP foci formation for the WT and *bdf1Δ* mutant cells. Red arrows indicate Rad52 foci. Over 100 cells were counted for each strain in each experiment (*n* = 3). A representative image is presented. D) Scheme showing gene targeting at two independent loci, *ISU1* and *ADH4*. The relative efficiency of gene integration was plotted (*n* = 4). E,F) Southern blot analysis and quantification of resection kinetics at indicated locations in the WT, *bdf1Δ or bdf1‐degron* cells (*n* = 3). Data analysis was performed by two‐way ANOVA and data were presented as mean ± SEM. G) ChIP‐qPCR showing the recruitment of RPA‐3xFLAG and Rad51‐3xFLAG at DSB ends in the WT and *bdf1Δ* cells (*n* = 3). The Signal was normalized to that of the corresponding “0” time point sample. H) Western blot showing Rad53 phosphorylation upon the HO‐induced DSB. Data presented in C, D, and G are mean ± S.D., *p*‐values are calculated using unpaired two‐tailed Student's *t*‐test, *p < 0.05; **p < 0.01; ***p < 0.001.

To probe how histone acetylation signal is integrated into the DNA damage response network, we took advantage of the yeast knockout collection and screened the non‐essential HAT and HDAC mutants as well as the strains lacking BRD‐ or BRD‐like proteins for cells with elevated DNA damage sensitivity (**Figure** **1**A). Interestingly, we found that, in addition to the *rtt109Δ* mutant, the BRD mutants *bdf1Δ* and *spt7Δ* are hypersensitive to multiple DNA‐damaging agents, such as camptothecin (CPT), phleomycin, or hydroxyurea (HU) (Figure S1, Supporting Information). Rtt109 is the acetyltransferase for H3K56 acetylation and is known to be implicated in DNA replication and maintenance of genome stability,^[^ ^27^, ^28^ ^]^ while Spt7 functions in the assembly of SAGA transcriptional regulatory complex.^[^ ^29^, ^30^ ^]^ In contrast, the role of Bdf1 in DNA damage response is largely unknown. Previous studies showed that Bdf1 is implicated in snRNA biogenesis, transcription, heterochromatin maintenance, recovery from replication fork breakage, and meiosis.^[^ ^25^, ^31^, ^32^, ^33^, ^34^ ^]^ Here, we focus on its roles in DNA repair and maintenance of genome stability.

### 2.2 Bdf1 Promotes DSB End Resection, Checkpoint Activation, and HR Repair

We confirmed the drug sensitivity for the *bdf1Δ* mutant in the JKM139 background. The defect was rescued by integrating a *BDF1‐3xFLAG* cassette at the endogenous locus (Figure 1B). Compared to the WT cells, the *bdf1Δ* mutant showed a reduced survival rate upon transient phleomycin or MMS treatment (Figure S2A, Supporting Information). Consistent with previous studies, *bdf1Δ* cells accumulated five times more spontaneous DNA lesions than the WT cells, as reflected by increased spontaneous Rad52‐YFP foci (Figure 1C).^[^ ^35^ ^]^ Thus, Bdf1 is crucial for response to both induced and spontaneous DNA damage. To test whether Bdf1 affects HR, we employed a recombination assay that monitors the integration rate of a *URA3* cassette at two independent loci (*ISU1* and *ADH4*) in the genome.^[^ ^36^ ^]^ At both loci, the relative integration efficiency was ≈30% in WT cells, while it decreased to less than 5% in the *bdf1Δ* mutant (Figure 1D), indicating an important role of Bdf1 in promoting HR.

Next, we examined the role of Bdf1 on DSB end resection using a haploid strain wherein a single DSB is generated at the *MAT*a locus on chromosome III by the HO endonuclease upon galactose induction (Figure 1E). The homologous donor sequences *HML* and *HMR* were deleted so that these cells cannot repair the DSB by HR.^[^ ^8^ ^]^ The resection kinetics was monitored using Southern blot analysis. Compared to the WT cells, the *bdf1Δ* mutant exhibited poor resection at both proximal ends and 10 or 28 kb distal ends (Figure 1E,F). This was not due to any shifts in the cell cycle (Figure S2B, Supporting Information). Accordingly, the loading of RPA and Rad51 at DSBs was abolished, and checkpoint activation reflected by Rad53 phosphorylation was deficient in *bdf1*Δ cells (Figure 1G,H).

To confirm the above results, we used the auxin‐inducible degron (AID) system to deplete Bdf1 temporarily.^[^ ^37^ ^]^ Endogenous Bdf1 was fused with a 3xmAID tag at the N‐terminus. This fusion protein was degraded within 30 min after the addition of 1 × 10^−3^ m IAA (Figure S3A,B, Supporting Information). As expected, the depletion of Bdf1 led to increased DNA damage sensitivity, elevated spontaneous DNA lesions, and compromised resection and checkpoint activation (Figure 1F; Figure S3C–F, Supporting Information). Thus, we conclude that Bdf1 is critical to promote DSB end resection, checkpoint activation, and HR repair.

### 2.3 The BRDs of Bdf1 Are Important for the DNA Damage Response and HR Repair

**Figure 2:**
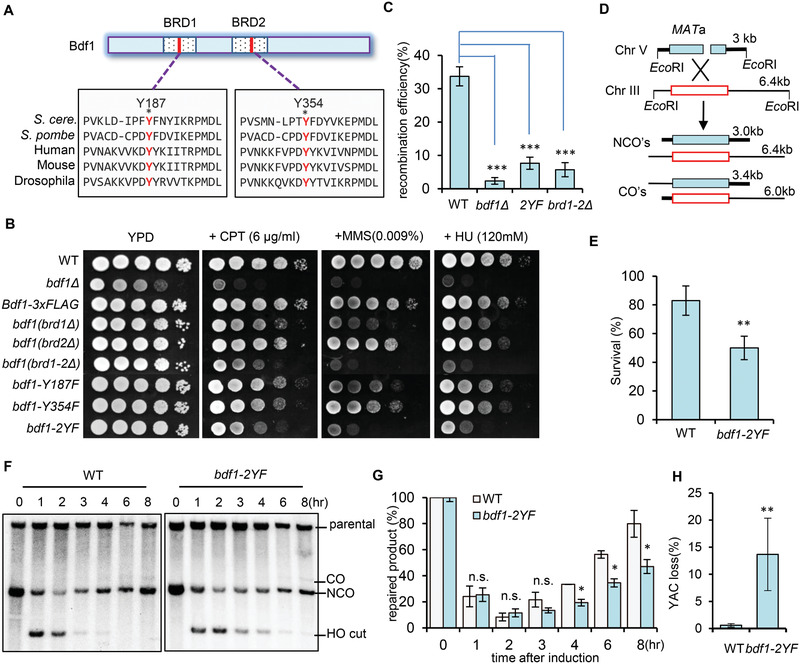
The bromodomains of Bdf1 are important for the DNA damage response and DSB repair by HR. A) Scheme showing the conserved motifs in the BRD domains of Bdf1 and its orthologs from indicated species. The two key residues, Y187 and Y354 (marked in red), are critical for maintaining the structure of BRDs. B) DNA damage sensitivity test for indicated strains at indicated drug concentrations. C) Plot showing the relative efficiency of gene integration at the *ISU1* locus in indicated strains (*n* = 4). D) Scheme showing the assay for ectopic recombination repair of the HO‐induced DSB. E) The survival rate of ectopic recombination for the WT and *bdf1‐Y2F* mutant (*n* = 4). F,G) Southern blot analysis and quantification of repair kinetics for the WT and the *bdf1‐2YF* mutant cells (*n* = 3). H) Plot showing the frequency of YAC loss in indicated cells (*n* = 3). Data in this figure are presented as mean ± S.D., *p*‐values are calculated using unpaired two‐tailed Student's *t*‐test, *p < 0.05, **p < 0.01, ***p < 0.001. n.s. means no significance.

The BET family protein Bdf1 has two conserved BRD motifs, BRD1 and BRD2 (**Figure** **2**A).^[^ ^25^, ^32^, ^34^ ^]^ The key residues Y187 in BRD1 and Y354 in BRD2 are essential for maintaining proper BRD structure.^[^ ^25^, ^32^, ^34^ ^]^ We evaluated the importance of BRDs in the DNA damage response. Disruption of BRD1 or BRD2 by deletion or point mutation (Y187F or Y354F) caused a moderate sensitivity to DNA damaging agents, while simultaneous disruption of both BRDs resulted in a hypersensitive phenotype (Figure 2B). Thus, the two BRD motifs act synergistically to promote DNA damage response. Notably, unlike the *bdf1Δ* null mutant, the *bdf1‐2YF* point mutation did not significantly affect the expression of genes involved in DSB repair or checkpoint signaling (Figure S4, Supporting Information). Therefore, we used the *bdf1‐2YF* point mutant as a separation‐of‐function mutant to study the role of Bdf1 in HR.

Next, we assessed the role of BRDs in HR using gene targeting. We found that recombination efficiency is significantly reduced in *bdf1‐2YF* cells, as seen in the *bdf1Δ* null mutant or the BRD deleting mutant (Figure 2C). To confirm this result, we used an ectopic recombination system in which a single HO‐induced DSB on chromosome V is repaired using the homologous sequence on chromosome III as a donor.^[^ ^38^ ^]^ Over 80% of WT cells completed the repair and survived, while only ≈50% of mutant cells survived (Figure 2D,E). Accordingly, the mutant repaired the break with slower kinetics (Figure 2F,G). Furthermore, we tested the role of BRDs on chromosome loss using a yeast system that carries an extra ≈320‐kb YAC.^[^ ^39^ ^]^ The dispensability of YAC allows us to assess the frequency of chromosome loss by monitoring the markers *URA3* and *HIS3* on the YAC.^[^ ^39^ ^]^ Compared to the WT cells, the *bdf1‐2YF* mutant exhibited a sharp increase (>30 fold) in chromosome loss (Figure 2H). Thus, the BRDs of Bdf1 are important for HR and genome stability.

Notably, unlike *bdf1Δ* null cells, the *bdf1‐2YF* point mutant only exhibited a moderate delay (about 2hrs) in resection than the WT cells (Figure S5A,B, Supporting Information). Consistently, the recruitment of the resection enzymes Sgs1, Dna2 and Exo1 were significantly impaired in the *bdf1‐2YF* mutant (Figure S5C–E, Supporting Information). Histone eviction or loss at DSB ends is required for proper DSB end resection and HR repair.^[^ ^36^, ^40^, ^41^, ^42^, ^43^, ^44^, ^45^, ^46^ ^]^ We noted that the histone H3 was lost at a comparable rate at DSB ends in the WT and *bdf1‐2YF* cells (Figure S5F, Supporting Information), suggesting that the defects in resection and HR repair in *bdf1‐2YF* cells are not due to any impairments in histone loss at DSB ends.

Notably, the increase of resection in *bdf1‐2YF* cells by overexpressing *EXO1* or by deleting *DOT1*, which is known to suppress resection by facilitating Rad9 recruitment,^[^ ^36^, ^47^ ^]^ was unable to rescue the HR defect (Figure S5G,H, Supporting Information). These results indicate that the resection delay may not be the only reason causing the HR defect in *bdf1*‐2*YF* cells. In support of this result, cells lacking the resection enzyme Sgs1, Exo1, or Fun30, exhibited a normal survival rate from the repair by ectopic recombination (Figure S5I, Supporting Information).^[^ ^8^, ^36^ ^]^ Therefore, we reasoned that the BRDs must play additional roles in HR.

### 2.4 The BRDs Bind Acetylated H4 In Vitro and In Vivo

**Figure 3:**
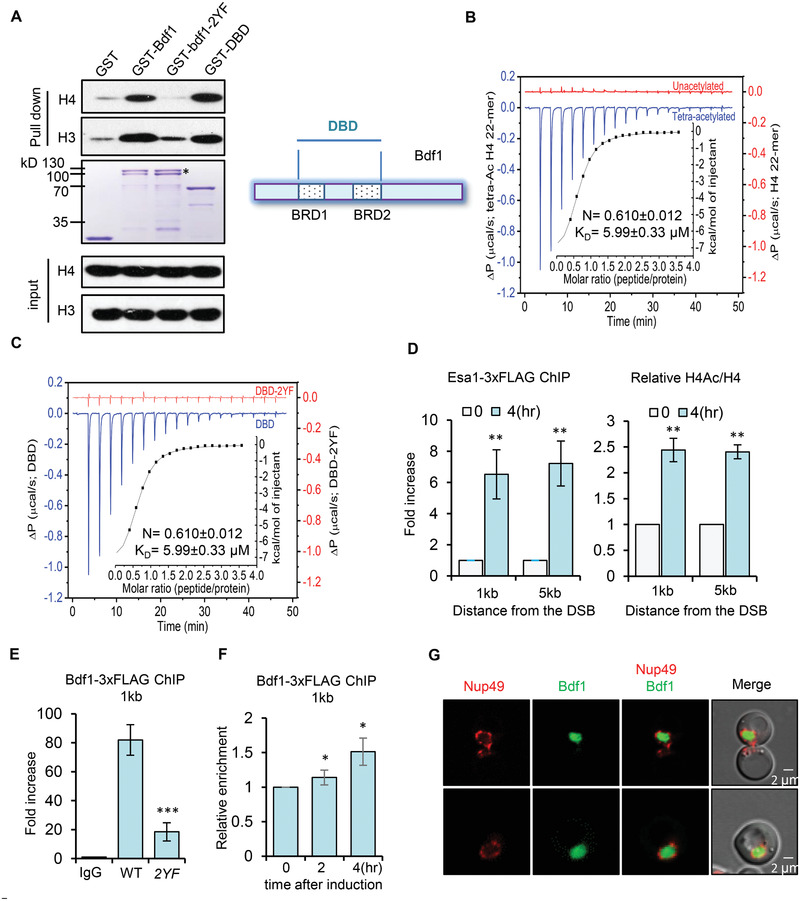
Bdf1 preferentially binds acetylated nucleosomes on chromatin. A) GST pull‐down assay showing the direct interaction between the WT or mutant Bdf1 and histone H4 or H3. GST‐tagged proteins (Bdf1, bdf1‐2YF, or DBD) immobilized on glutathione agarose beads were used to capture the oligomer nucleosomes preassembled with ssDNA and purified *Xeponus* histones. The amount of GST‐tagged proteins used for experiments is indicated by Coomassie blue staining. * denotes the full‐length GST‐Bdf1. The scheme shows the DBD (double bromodomains and the linker area) of Bdf1. B) Isothermal titration calorimetry (ITC) assay measuring the binding of GST‐DBD (50 × 10^−6^ m) to acetylated or nonacetylated H4 peptide (1–22 aa, 1 × 10^−3^ m). C) ITC profiles showing the binding of acetylated H4 peptide (1–22 aa, 1 × 10^−3^ m) to GST‐DBD or GST‐DBD‐2YF(50 × 10^−6^ m). Δ*P*, differential power. D) ChIP‐qPCR showing the relative enrichment of Esa1‐3xFLAG and H4Ac at DSB ends (*n* = 3). E) ChIP showing the binding of Bdf1‐3xFLAG or bdf1‐2YF‐3xFLAG at the break site before DSB induction (*n* = 3). F) ChIP indicating the relative enrichment of Bdf1‐3xFLAG at DSBs following DSB induction (*n* = 3). The relative fold increase in D and F was calculated by normalizing the ChIP signal to that of the corresponding “0” time point sample. The enrichment of Bdf1 in E was calculated by normalizing the ChIP signals to the control sample (IgG). Data are presented as mean ± S.D., *p*‐values are calculated using unpaired two‐tailed Student's *t*‐test, **p < 0.05, **p < 0.01, ***p < 0.001. G) Microscopy observation of the nuclear localization of Bdf1‐GFP. Nuclear envelop is indicated by Nup49‐mCherry.

BRD motifs preferentially bind acetyl‐lysines.^[^ ^25^, ^32^, ^48^, ^49^, ^50^, ^51^ ^]^ We tested whether Bdf1 binds nucleosomes. Oligomer nucleosomes were assembled in vitro using purified histone octamers of *Xenopus* oocytes with pG54E plasmid DNA as described.^[^ ^52^ ^]^ Recombinant GST‐Bdf1, GST‐bdf1‐2YF, or GST‐DBD (which contains the two BRDs and the linker region) protein was immobilized to glutathione agarose beads to carry out pull‐down assay. We observed that GST‐Bdf1 but not the GST‐bdf1‐2YF mutant protein strongly associates with nucleosomes (**Figure** **3**A). Notably, GST‐DBD alone was sufficient to bind nucleosomes with an affinity comparable to WT Bdf1. Thus, BRDs play a critical role in mediating the association of Bdf1 with nucleosomes (Figure 3A).

Next, we compared the affinity of GST‐DBD and GST‐DBD‐2YF for non‐acetylated or fully acetylated (K5, K8, K12, and K16) versions of H4 peptide (1‐22 aa) using isothermal titration calorimetry (ITC). We noted that GST‐DBD bound the acetylated peptide with an affinity of ≈6 × 10^−6^ m, while it did not bind the non‐acetylated H4 peptide (Figure 3B). In contrast, GST‐DBD‐2YF mutant protein exhibited no binding even for the acetylated H4 (H4Ac) (Figure 3C). These results demonstrate that Bdf1 binds nucleosomes, but preferentially the acetylated H4, via its BRD motifs.

In line with previous studies, we detected the recruitment of Esa1, the catalytic subunit of the NuA4 acetyltransferase complex, and the relative enrichment of H4ac to H4 at the DSB ends (Figure 3D).^[^ ^53^ ^]^ Notably, we detected robust binding of Bdf1‐3xFLAG at the HO cut site before DSB induction by ChIP, and the binding was severely impaired in *bdf1‐2YF* cells (Figure 3E), indicating a critical role of BRDs in mediating Bdf1 chromatin association. This was not caused by any alterations in the protein levels of Bdf1 or H4ac (Figure S6A,B, Supporting Information). However, we only observed a modest increase in Bdf1 enrichment upon DSB induction (Figure 3F). This is likely due to its chromatin‐bound nature. Indeed, the Bdf1‐GFP fusion protein fully localizes in the nucleus, as it is encircled by Nup49‐mCherry, a marker of the nuclear envelope (Figure 3G). Moreover, we noted that Bdf1 remains associating with the chromatin regardless of the DNA damage (Figure S6C, Supporting Information). Together, these results indicate that Bdf1 preferentially binds acetylated chromatin via its BRDs in cells.

### 2.5 Bdf1 Physically Interacts with RPA and Facilitates RPA Loading at DSBs

**Figure 4:**
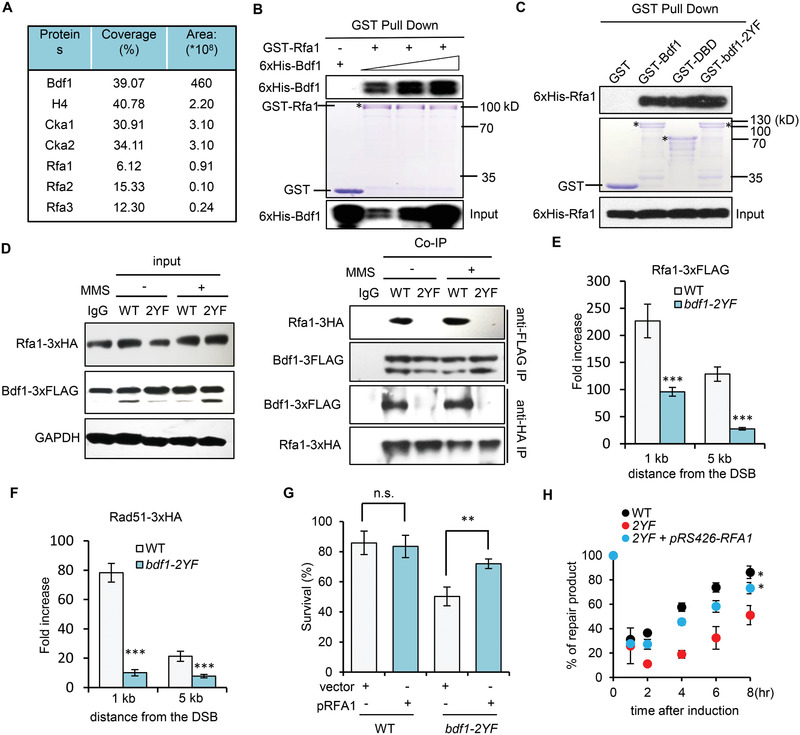
Bdf1 interacts with RPA and stimulates RPA loading in vivo. A) Mass spectrum analysis identified a list of proteins associating with the DBD motif of Bdf1. For each protein, the percentage of peptide sequence covered is indicated. The area represents the abundance of each protein among all identified proteins. B) GST pull‐down assay showing the interaction between GST‐Rfa1 and 6xHis‐Bdf1 in a dose‐dependent manner. GST was used as a negative control. The amount of GST‐Rfa1 used for experiments is indicated by Coomassie blue staining (middle panel). * denotes the full‐length GST Rfa1. The associated 6xHis‐Bdf1 was detected by Western blot with an anti‐His antibody (upper panel). C) GST pull‐down assay showing the interaction between GST‐Bdf1, GST‐DBD, or GST‐bdf1‐2YF and 6xHis‐Rfa1. GST was used as a negative control. The amount of WT or mutant GST‐Bdf1 protein used for experiments is indicated by Coomassie blue staining (middle panel). * denotes the full‐length GST tagged WT or mutant Bdf1 protein. The associated 6xHis‐Bdf1 was detected by Western blot with an anti‐His antibody (upper panel). D) Co‐immunoprecipitation and Western blot analysis of the interaction between Rfa1‐3xHA and Bdf1‐3xFLAG or bdf1‐2YF‐3xFLAG. Cells with or without MMS (0.1%, 90 min) were used for experiments. Benzonase (500 U mL^−1^) was included in the cell lysates to digest DNA or RNA. E,F) ChIP‐qPCR analysis of the loading of Rfa1‐3xFLAG or Rad51‐3xFLAG at 1 or 5 kb upstream of the HO cut 4 hours after DSB induction in indicated cells (*n* = 3). The Signal was normalized to that of the corresponding “0” time point sample. G) Survival rate of ectopic recombination in indicated cells (*n* = 3). The data in E, F, and G are presented as mean ± S.D., *p*‐values are calculated using unpaired two‐tailed Student's *t*‐test, **p < 0.01 ***p < 0.001. n.s.: no significance. H) Quantification of repair kinetics of ectopic recombination for indicated strains (*n* = 3). Data analysis was performed by two‐way ANOVA and data were presented as mean ± SEM. *p < 0.05.

To address how Bdf1 may regulate the downstream HR repair, we used recombinant GST‐DBD (containing BRD1, BRD2, and the linker region) as a bait to capture the DBD‐associated proteins. Among the top hits, we found all three subunits of the RPA complex and the proteins known to associate with Bdf1, such as H4, Cka1, and Cka2^[^ ^32^, ^48^, ^49^, ^50^, ^54^ ^]^ (**Figure** **4**A). Given the critical function of RPA in DNA repair and recombination, we tested the interaction between RPA and Bdf1 by pull‐down assay. We observed that GST‐Rfa1 interacted with 6xHis‐Bdf1 protein in a dose‐dependent manner (Figure 4B). Notably, both GST‐DBD and GST‐bdf1‐2YF mutant proteins can bind Rfa1, suggesting that BRD1 and BRD2 themselves are not required for the interaction with RPA in vitro (Figure 4C). Consistently, Bdf1‐3xFLAG is associated with Rfa1‐3xHA in vivo regardless of the DNA damage. However, this association was abolished in the *bdf1*‐*2YF* mutant (Figure 4D). Thus, this interaction likely occurs in the context of chromatin, given that the BRDs are required for Bdf1 binding to chromatin. Notably, the loading of RPA and Rad51 at 4hr after the break induction was significantly impaired at DSBs in *bdf1*‐*2YF* cells (Figure 4E,F). This was not due to any reduction in the protein levels (Figure S6A, Supporting Information). Thus, the H4Ac‐dependent Bdf1 association with chromatin is important for proper RPA loading at DSBs, which is likely achieved via a direct Bdf1–RPA interaction.

This could be particularly meaningful for RPA loading on the chromatin context since it is known that a significant fraction of histones remain bound to ssDNA at DSBs that may impede the loading and spreading of RPA and Rad51 along ssDNA.^[^ ^36^, ^41^, ^42^, ^43^, ^45^, ^55^ ^]^ We asked whether overexpression of RPA could bypass the requirement of BRDs in promoting HR repair. Indeed, overexpression of RPA on a high‐copy plasmid in the *bdf1*‐*2YF* mutant significantly increased the survival rate and repair kinetics of ectopic recombination (Figure 4G,H), yet it did not cause a noticeable increase in resection (Figure S7A–C, Supporting Information). Consistently, the expression of excessive RPA fully restored Rad51 loading (Figure S7D, Supporting Information). Thus, the HR defect in *bdf1*‐*2YF* cells appears mainly due to defective RPA loading. Together, these results suggest that the H4Ac‐mediated Bdf1 binding on chromatin is important to promote RPA loading and HR repair.

### 2.6 The Bdf1–RPA Interaction Facilitates HR Repair in a Manner Epistatic to *bdf1‐2YF*

**Figure 5:**
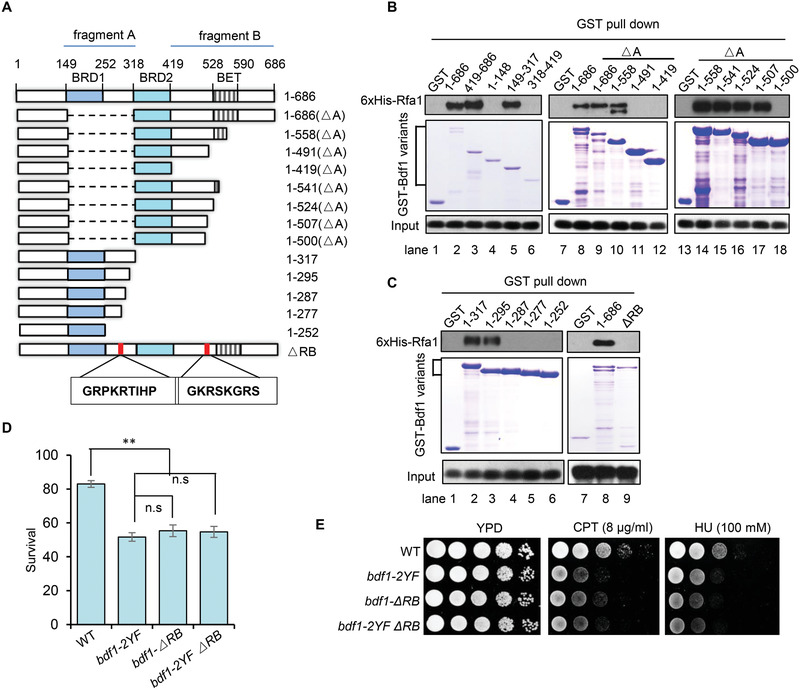
Disruption of Bdf1–RPA interaction results in an HR defect epistatic to the *bdf1‐2YF* mutant. A) Scheme showing the full‐length or truncated Bdf1 proteins used for pull‐down assays. Dash lines represent deleted regions, while the open bars represent truncated Bdf1 proteins. The motifs BRD1, BRD2, and BET are marked. The precise positions for these motifs or the truncation proteins are indicated. The RPA‐interacting motifs of Bdf1 are shown in the box. B,C) GST pull‐down assay showing the interaction between 6xHis‐Rfa1 and the WT or truncated GST‐Bdf1 proteins. GST was used as a negative control. The amount of WT or truncated GST‐Bdf1 protein used for experiments is indicated by Coomassie blue staining of SDS‐PAGEs (middle panel). The associated 6xHis‐Rfa1 was detected by Western blot (upper panel). D) The survival rate of DSB repair by ectopic recombination in indicated strains. Data analysis was performed by one‐way ANOVA followed by Turkey post‐hoc test and data were presented as mean ± SEM. *n* = 3. **p < 0.01, n.s.: no significance. E) DNA damage sensitivity test for indicated strains to CPT or HU.

To determine the Bdf1 domains that interact with RPA, we expressed a serial of GST‐tagged full‐length or truncated Bdf1 proteins and tested their associations with 6xHis‐Rfa1.We found that Bdf1 interacts with Rfa1 via two fragments independently. One is between residues 149–317 (fragment A) that contains BRD1 and the linker region, and the other is between residues 419–686 (fragment B) that covers the C‐terminal end after BRD2 (**Figure** **5**A,B, lane 1–6). To dissect the key residues in each of the fragments, we constructed a serial of Bdf1 truncations and examined their associations with RPA. In the absence of fragment A, the mutant protein containing residues1‐558 can interact with RPA, while the one containing residues 1–491 or less failed to do so, suggesting that the motif between 491–558 is required to mediate the interaction (Figure 5B, lane 7–12). A more detailed analysis revealed that the residues between 500 and 507 (located upstream of the BET domain) are required for the interaction (Figure 5B, lane 13–18). Similarly, in the absence of fragment B, the truncation protein containing residues 1–317 or 1–295 can bind RPA, while the one with residues 1–287 or less can no longer bind RPA, suggesting that the residues between 287 and 295 (located in the linker region between BRD1 and BRD2) are required for the interaction (Figure 5C, lane 1–6). Indeed, simultaneous deletion of both segments (500‐507aa and 287–295 aa, bdf1‐ΔRB) abolished the interaction between Bdf1 and RPA (Figure 5C, lane 7–9). Importantly, disruption of this interaction (*bdf1‐ΔRB*) impaired DSB repair by ectopic recombination to a similar extent as seen in the *bdf1‐* *2YF* single mutant or the *bdf1‐2YFΔRB* double mutant (Figure 5D), indicating that *bdf1‐ΔRB* and *bdf1‐2YF* mutations are epistatic in promoting HR repair. Consistently, the two mutants exhibited similar resistance to CPT or HU (Figure 5E).

Together, our results suggest a model that in response to DSBs, Bdf1 binds the DNA damage‐induced H4Ac on chromatin via BRD motifs; meanwhile, Bdf1 physically interacts with RPA via the linker and the C‐terminal regions. These interactions facilitate efficient RPA loading on exposed ssDNA with histones, leading to proper Rad51 loading and HR repair.

### 2.7 Human TAF1 Promotes DNA End Resection and HR Repair

**Figure 6:**
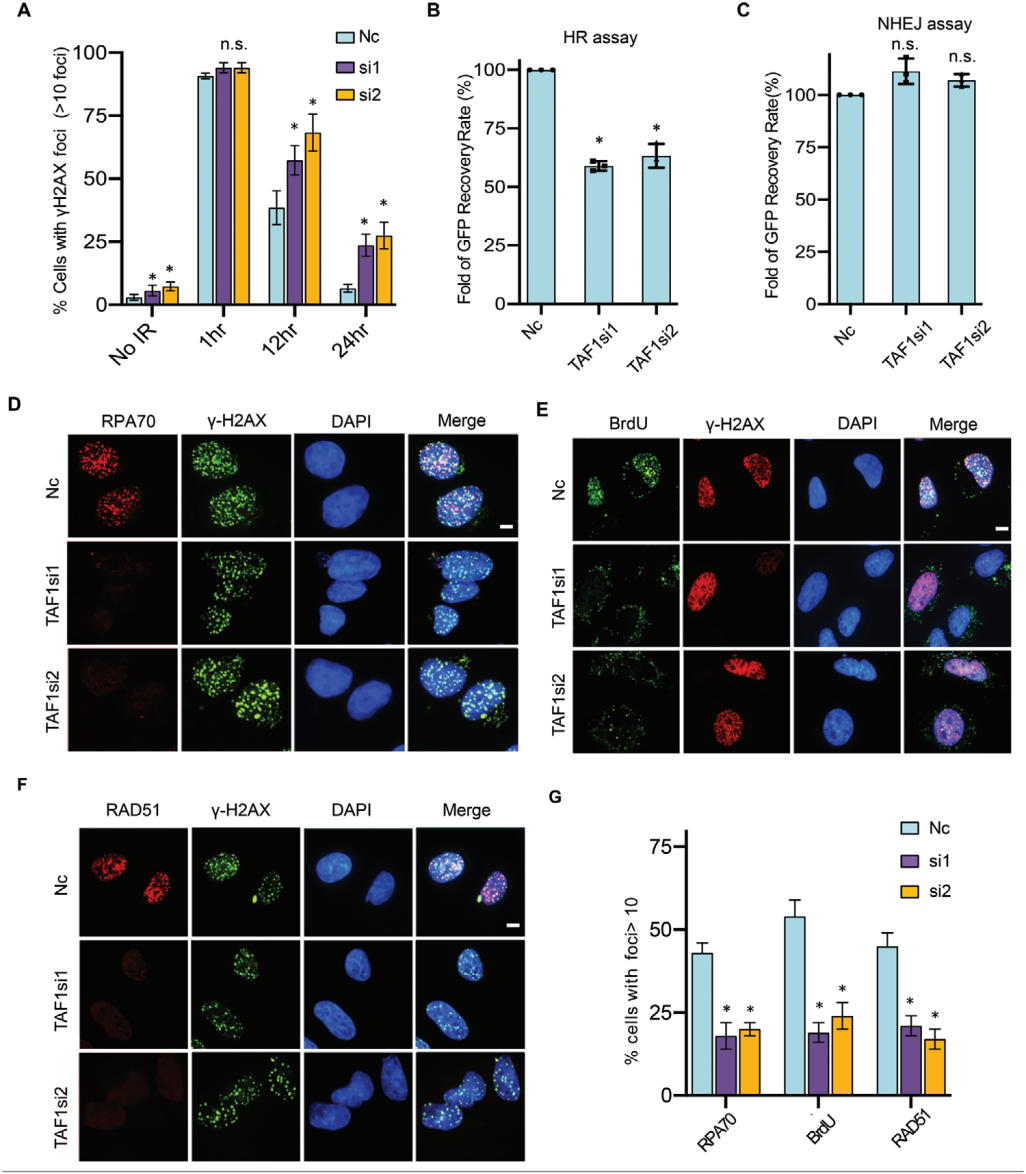
Human TAF1 promotes DSB end resection, RPA and RAD51 loading, and HR repair. A) DSB repair kinetics in TAF1 knockdown cells. Quantification of *γ*H2AX foci number along with the recovery post ionizing radiation (*n* = 3). B,C) Plots showing the efficiency of DSB repair by HR and NHEJ, respectively, in TAF1 knockdown cells (*n* = 3). D) Representative images of immunostaining of RPA70, E) BrdU, F) RAD51 foci in TAF1 knockdown cells. Scale bar: 10 µm. G) Quantification of (D–F) are average values of three independent experiments. Nearly 100 cells were counted for each experiment. Data were analyzed by Students’ *t*‐test and were presented as mean ± SD; *p < 0.05. n.s.: no significance

Bdf1 corresponds to the C‐terminal half of TAF1 in mammals.^[^ ^26^ ^]^ TAF1 also harbors two BRDs with 26.9% identity or 45.3% similarity with that of Bdf1 in peptide sequence (Figure S8A,B, Supporting Information). We asked whether TAF1 plays a similar role in human cells. Notably, depletion of TAF1 by RNAi in MCF7 cells rendered hypersensitive to multiple DNA damaging agents, including CPT, IR, and the PARP inhibitor AZD2281 (Figure S9A–D, Supporting Information). We observed that TAF1‐depleted U2OS cells accumulated elevated levels of spontaneous *γ*H2AX foci (**Figure** **6**A; Figure S9E,F, Supporting Information). Importantly, after IR treatment, TAF1 knockdown cells exhibited slower kinetics in removing *γ*H2AX compared to the WT cells, implying that TAF1 promotes the repair of IR‐induced DNA damage (Figure 6A; Figure S9F, Supporting Information). Next, we employed the *Sce*I‐induced DR‐GFP/EJ5‐GFP reporter system to measure the HR and NHEJ efficiency.^[^ ^56^ ^]^ Depletion of TAF1 significantly impaired the HR repair but not NHEJ (Figure 6B,C). Consistently, RPA loading and ssDNA generation were impaired in TAF1‐depleted cells, as reflected by diminished RPA foci and less BrdU staining (Figure 6D,E,G). Consequently, RAD51 loading was significantly reduced (Figure 6F,G). In contrast, the recruitment of upstream proteins, such as MDC1 and BRCA1, and the NHEJ protein 53BP1 remained unaffected (Figure S10A–D, Supporting Information). These results indicate that TAF1 promotes end resection, loading of RPA and RAD51, and HR repair.

### 2.8 The BRD Motifs of TAF1 Are Critical for Its Roles in HR

**Figure 7:**
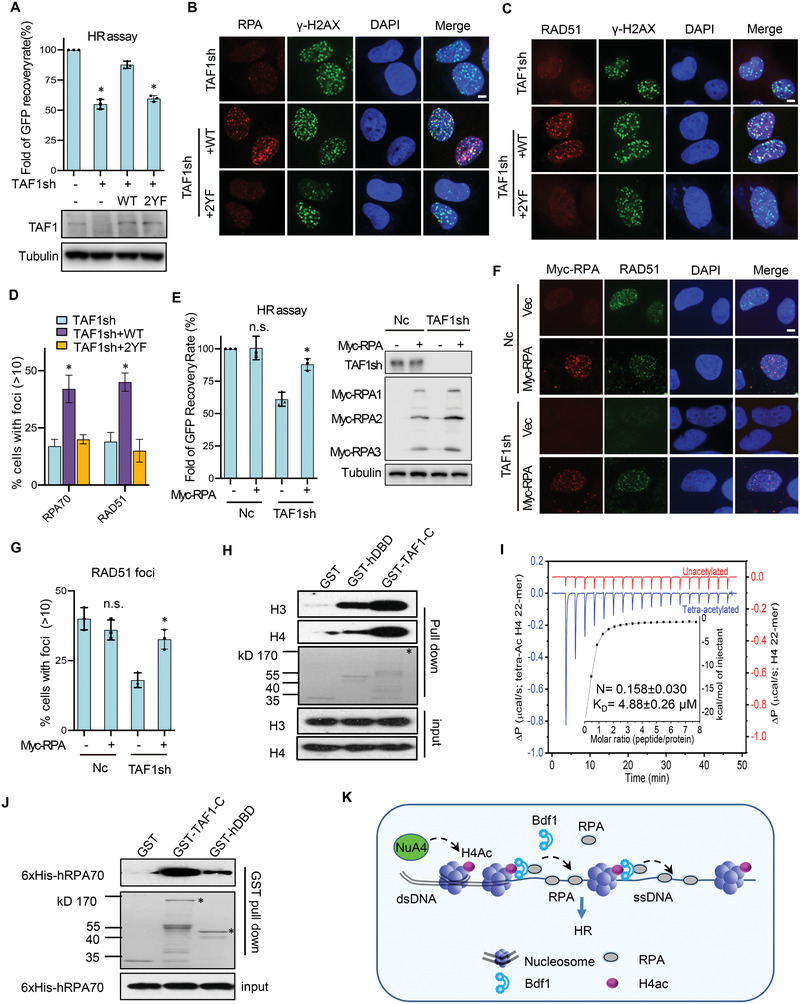
TAF1 BRDs are important for the loading of RPA and RAD51 and the repair by HR. A) Plot showing the HR efficiency in TAF1‐depleted U2OS cells complemented with a plasmid‐borne WT *TAF1* gene or *TAF1‐2YF* mutant allele (*n* = 3). The WT and TAF1 knockdown cells were used as controls. The levels of TAF1 in these cells are indicated by Western blot. B,C) Immunostaining of the DNA damage‐induced RPA and RAD51 foci in indicated cells. Scale bar: 10 µm. D) Quantification of (B,C) is the average of three independent experiments (*n* = 3). Nearly 100 cells were counted for each experiment. E) The HR efficiency in the WT or TAF1‐depleted U2OS cells with or without the overexpression of Myc‐RPA(*n* = 3). The protein levels of TAF1 and subunits of the RPA complex are indicated by Western blot. F,G) Immunostaining and quantification of DNA damage‐induced RAD51 foci formation in the WT or TAF1‐depleted U2OS cells with or without the overexpression of Myc‐RPA(*n* = 3). Scale bar: 10 µm. Plotted are the average values of three independent experiments. About 100 cells were counted for each experiment. H) GST pull‐down assay showing the interaction between preassembled nucleosomes and GST‐TAF1‐C or GST‐hDBD. The oligomer nucleosomes were assembled with ssDNA and purified *Xeponus* histones. GST was used as a negative control. * denotes GST‐TAF1‐C. I) ITC profiles showing the binding of TAF1 DBD (20 × 10^−6^ m) to acetylated or nonacetylated H4 peptide (500 × 10^−6^ m). J) GST pull‐down assay showing the interaction between GST‐TAF1‐C or GST‐hDBD and 6xHis‐RPA70. GST was used as a negative control. The amount of GST‐TAF1‐C and GST‐hDBD protein used for experiments is indicated by Coomassie blue (middle panel). * denotes GST‐TAF1‐C or GST‐hDBD. The associated 6xHis‐hRPA70 was detected by Western blot (upper panel). K) A working model for Bdf1. In response to DSBs, histone H4 around the break sites becomes acetylated by the acetyltransferase NuA4. Bdf1 binds the DNA damage‐induced H4Ac via the BRD motifs, leading to its chromatin recruitment. Meanwhile, Bdf1 physically interacts with RPA via the linker and C‐terminal domains, and this interaction facilitates RPA loading in the chromatin context and the subsequent HR repair. Data in this figure are presented as mean ± S.D., *n* = 3, *p*‐values are calculated using unpaired two‐tailed Student's *t*‐test, *p < 0.05, n.s.: no significance.

Next, we tested whether disruption of TAF1 BRDs by mutating of the conserved residues (Y1417 in BRD1 and Y1540 in BRD2, *TAF1‐2YF*, Figure S8A,B, Supporting Information) could affect RPA loading and HR repair. In TAF1 knockdown cells, complementation of a plasmid bearing a WT *TAF1* gene rescued the HR defect, while introducing a *TAF1‐2YF* mutant allele failed to do so (**Figure** **7**A). Similarly, the defects in RPA and Rad51 loading in TAF1‐depleted cells can only be rescued by introducing the WT *TAF1* but not the *TAF1‐2YF* allele (Figure 7B–D). Thus, the BRDs are essential for TAF1 to fulfill its function in promoting the loading of RPA and RAD51 and the repair by HR.

TAF1‐mediated phosphorylation of p53 on Thr55 is important to inactivate p53, such that in TAF1 knockdown cells, activated p53 activity causes upregulation of p21, which inhibits the cyclin‐dependent protein kinases required for the G1‐to‐S phase transition, leading to G1 cell cycle arrest (Figure S10E,F, Supporting Information).^[^ ^57^, ^58^ ^]^ However, these changes can be rescued by introducing either the WT *TAF1* or the *TAF1‐2YF* allele (Figure S10E,F, Supporting Information), suggesting that the functions of TAF1 BRDs in promoting resection and HR repair do not involve altered p53 or p21 activities.

Notably, the expression of excessive human RPA in TAF1‐depleted cells also restored the efficiency of HR repair and RAD51 recruitment, as seen in yeast (Figure 7E–G). Thus, the role of TAF1 in facilitating RPA loading is important for efficient HR repair in human cells. Together, we establish a conserved role for Bdf1 and TAF1 in promoting RPA loading and HR repair via their BRD motifs.

### 2.9 TAF1 Binds Nucleosomes and hRPA In Vitro

To test whether human TAF1 could act by a similar mechanism as Bdf1, we expressed the C‐terminal half of TAF1 (GST‐TAF1‐C, which corresponds to the yeast Bdf1) and GST‐hDBD (which contains BRD1, BRD2 and the linker region) and tested their binding abilities for nucleosomes by pull down. We observed that GST‐TAF1‐C but not GST itself associated with nucleosomes. Similarly, hDBD alone can bind nucleosomes, albeit with a reduced affinity (Figure 7H). As expected, hDBD bound the H4Ac peptide with a Kd of ≈ 5 × 10^−6^ m, but it did not bind the non‐acetylated H4 peptide (Figure 7I). Thus, like Bdf1, TAF1 also preferentially binds H4Ac via its BRDs. Similarly, we found that GST‐TAF1‐C and to a lesser extent GST‐hDBD directly interact with 6xHis‐hRPA70 (Figure 7J). These results raise the possibility that Bdf1 and TAF1 may act by a similar mechanism in promoting DSB repair by HR via binding acetylated chromatin and interacting with RPA.

## 3 Discussion

### 3.1 Bdf1/TAF1 Links the HR Pathway to Histone Acetylation

Histone acetylation plays an essential role in DSB repair and maintenance of genome stability, but the underlying mechanism remains partially understood. Here, we identified that the yeast BRD protein Bdf1 and its human counterpart TAF1 play a conserved role in linking HR repair to histone acetylation in a manner dependent on their BRD motifs. We provided evidence that Bdf1 preferentially binds the DNA damage‐induced histone H4 acetylation (H4Ac) via the BRD motifs, leading to its chromatin recruitment (Figure 7K). On the other hand, Bdf1 physically interacts with RPA via the linker and the C‐terminal region and this interaction appears to facilitate or stabilize RPA loading in the chromatin context where ssDNA is bound by dispersed histones. These events lead to efficient Rad51 loading and HR repair (Figure 7K). As a result, disruption of BRD motifs impaired the chromatin‐recruitment of Bdf1, the loading of RPA and Rad51, and the repair by HR, resulting in increased genome instability and DNA damage sensitivity. Notably, our results suggest that TAF1 promotes the loading of RPA and Rad51 and DSB repair by HR by a similar mechanism. Thus, Bdf1/TAF1 links RPA loading and HR repair to chromatin acetylation in both yeast and human. Consistently, Bdf1 was shown to be required for spontaneous or gap‐induced sister chromatid recombination in a fashion epistatic to H4 acetylation.^[^ ^59^ ^]^

### 3.2 The Assembly of RPA on ssDNA in the Context of Chromatin

RPA has a high affinity for ssDNA and plays essential roles in all DNA transactions. Single‐molecule studies revealed that RPA binding on ssDNA is highly dynamic, and it can rapidly diffuse within the bound DNA ligand and quickly exchange between the free and ssDNA‐bound states.^[^ ^60^, ^61^, ^62^, ^63^, ^64^ ^]^ The binding dynamics and cellular functions of RPA appear to rely on both its high affinity for ssDNA and its ability to interact with different enzymes or proteins.^[^ ^64^ ^]^ Indeed, recent studies showed that the RPA‐interacting protein Rtt105 is required to stimulate RPA loading at replication forks in the chromatin context.^[^ ^65^ ^]^

At DSB ends, a significant fraction of histones remain bound to the 3’‐ssDNA generated by resection within 4 hours after break induction.^[^ ^36^, ^40^, ^43^, ^45^, ^55^ ^]^ Indeed, in vitro evidence showed that histone octamers could form a “nucleosome‐like” structure on ssDNA.^[^ ^66^, ^67^, ^68^ ^]^ How RPA assembles and diffuses on the ssDNA with histone barriers remains to be a question. Our results revealed that the interaction between RPA and the chromatin‐bound Bdf1/TAF1 might play a unique role in facilitating RPA loading or spreading in this context. As a result, disruption of Bdf1–RPA interaction impaired HR, while overexpression of RPA largely rescued the defects of Rad51 loading and HR repair in both *bdf1‐2YF* and TAF1‐depleted cells. However, it remains to be determined whether RPA loading during DNA replication or transcription is also regulated by Bdf1. Future studies should aim to address this question at a global level. Our results do not exclude the possibility that the moderately delayed resection in *bdf1‐2YF* cells might also contribute partially to the HR defect. How Bdf1 and TAF1 facilitate DSB end resection remains to be elucidated. Interestingly, it has been reported that TAF1 associates with the central resection enzyme MRE11 via its C‐terminus in *Arabidopsis*.^[^ ^69^ ^]^ Indeed, our unpublished results showed that yeast Bdf1 and human TAF1 can also interact with Mre11 in vitro (not shown), suggesting the conservation of this interaction. It is likely that this interaction facilitates Mre11 loading at DSB ends, thereby promoting DNA end resection.

### 3.3 The Interaction between Bdf1 and RPA

Our results showed that Bdf1 binds acetylated chromatin via the two BRD motifs and interacts with RPA through the linker and the C‐terminal motif. Structural studies of human TAF1 have revealed that the linker between BRD1 and BRD2 forms a relatively disordered region,^[^ ^48^ ^]^ providing potential interfaces to interact with other proteins. One of the RPA‐interacting motifs in Bdf1, which appears to form a conserved hydrophilic region, is located in this region (Figure S11A,B, Supporting Information). Interestingly, we found that Bdf1 itself can form an oligomer complex in vitro (not shown). Therefore, the interaction between Bdf1, acetylated chromatin and RPA in cells could be more complex than anticipated. How the interaction between Bdf1 and RPA is regulated in cells remains to be investigated. Although Bdf1 can act as a component of the SWR complex that deposits the histone variant H2AZ,^[^ ^70^, ^71^ ^]^ the role of Bdf1 in HR is independent of the SWR complex since deletion of *SWR1* does not impair the survival rate of ectopic recombination (Figure S5I, Supporting Information). Notably, *bdf1Δ* cells exhibited more severe defects than *bdf1‐2YF* cells in drug resistance or DNA end resection (Figures 1E,F and 2B; Figure S5A,B, Supporting Information), suggesting that the regions beyond BRDs also contribute to the DNA damage response.

### 3.4 The Roles of BRD Proteins in DSB Repair and Genome Stability

Two recent screens identified that over half (24 out of 40) of human BRD proteins are involved in DSB repair, especially the HR pathway, underscoring the essentiality of BRD proteins in preserving genome integrity.^[^ ^22^, ^23^ ^]^ TAF1 was also identified in the screen,^[^ ^23^ ^]^ but it does not form visible foci at DNA lesions.^[^ ^22^ ^]^ This is likely due to the chromatin‐bound nature, so its accumulation at DNA damage sites is difficult to detect. Currently, a complete understanding of how histone acetylation‐BRD protein functions to promote DNA repair and genome stability remains elusive. Our study provides an insight into how chromatin‐based mechanisms dictate DSB repair and maintain genome stability. Notably, TAF1 is highly mutated in cancers and is associated with cancer progression.^[^ ^72^, ^73^, ^74^ ^]^ Our understanding of the role of TAF1 in the DNA damage response and repair may assist in developing new therapeutic strategies targeting cancer.

## 4 Experimental Section

### Yeast Strains, Human Cell Culture, and Reagents

Strains used in this study were derivatives of JKM139 (*ho MATa hml::ADE1 hmr::ADE1 ade1‐100 leu2‐3112 trp1::hisG’ lys5 ura3‐52 ade3::GAL::HO*) or tGI354 (*MATa‐inc arg5,6::MATa‐HPH ade3::GAL::HO hmr::ADE1 hml::ADE1 ura3‐52*). All yeast strains used in this study are listed in Table S1 in the Supporting Information. Yeast strains were constructed using standard genetic manipulation. Point mutants were verified by sequencing.

Human embryonic kidney 293T cells (ATCC, CRL‐11268), human osteosarcoma cell line U2OS (ATCC, HTB‐96) and human breast cancer cell line MCF7 (ATCC, HTB‐22) were maintained in Dulbecco's modified Eagle's medium (DMEM) supplemented with 10% fetal bovine serum (FBS) and penicillin/streptomycin (P/S) at 37 °C with 5% CO2. U2OS DR‐GFP and EJ5‐GFP cells used for DNA repair reporter assay were maintained in DMEM without pyruvate, supplied with 10% FBS and P/S. Plasmid CMV‐HA‐hTAF1 was from Addgene (# 17 997). TAF1‐2YF was generated by site‐directed mutagenesis and confirmed by sequencing. shRNA targeting the 3’ UTR region of TAF1 mRNA was from Sigma (TRCN0000280259). HCT116 cells used for cell cycle analysis were maintained in RPMI‐1640 medium supplemented with 10% FBS and P/S at 37 °C with 5% CO2.

### Drug Sensitivity Test

Yeast DNA damage sensitivity was determined using a spotting assay. Overnight yeast cultures were diluted to serials of different concentrations. 5 µL of each diluted culture was spotted onto YPD plates with indicated DNA‐damaging agents. Plates were incubated at 30 °C for 3–4 days before taking pictures. For the test in human cells, MCF7 cells transfected with indicated siRNA were seeded 4000 cells per well in 96 well plates. 12 h later, cells were treated with various DNA‐damaging agents at indicated concentrations and were incubated at 37 °C for three days. The cell viability was analyzed using CCK‐8 kit (Dojindo). Data were presented as the average of three independent experiments. Error bar denotes the standard error of the mean (SEM).

### Fluorescence Microscopy

For yeast, live cells were examined using an Olympus BX53 fluorescence microscope with a 100 x oil immersion objective lens and a YFP filter. Fluorescent images were captured using an Olympus DP80 digital camera and analyzed using the Olympus Cellsens software. The percentage of cells carrying Rad52‐YFP foci was calculated after analyzing three independent experiments. Approximately 100 cells were counted for each experiment.

To examine the foci formation for human proteins, the U2OS cells grown on coverslips were treated with 5 Gy ionizing radiation (IR) followed by 3 h recovery. Cells were then fixed in 4% paraformaldehyde (PFA) for 15 min and permeabilized in 0.5% Triton X100 solution for 5 min. After blocked by 5% goat serum, cells were incubated with primary antibodies overnight and subsequently secondary antibodies for 1 h. Coverslips were then mounted using DAPI containing anti‐fade. To detect ssDNA generation, BrdU was incorporated into cells overnight before IR treatment. Right before fixation, cells were prepermeabilized with 0.1% Triton‐X100 solution on ice for 5 min. Images were captured using an Olympus DP80 digital camera. Images were processed using Olympus Cellsens software. The percentage of cells carrying indicated foci was calculated after analyzing three independent experiments. Approximately 100 cells were counted for each sample. Antibodies used for immunofluorescent staining were as follows: Mouse Anti‐γ‐H2AX (05‐636) antibody was purchased from Millipore, rabbit anti‐RPA70 and rabbit anti‐γ‐H2AX (#9718) antibodies were obtained from Cell Signaling Technology. Rabbit anti‐RAD51 (GTX100469) antibody was bought from GeneTex. Mouse anti‐BRCA1 (D9) antibody was purchased from Santa‐Cruz. Rabbit anti‐53BP1 (NB100‐304) antibody was from Novus Biologicals, and mouse anti‐BrdU (555 627) antibody was from BD Pharmingen.

### Analysis of DSB End Resection and Ectopic Recombination

To measure resection kinetics, DSB was induced by adding of 2% galactose to the log phase (1 × 10^7^ mL^−1^) yeast cells grown in the preinduction medium (YP‐Raffinose). Samples were collected at 0, 1, 2, 4, 6, 8, 10, and 12 h after DSB induction. Genomic DNA was extracted following the standard phenol‐chloroform method. Purified genomic DNA was digested with *E*coRI followed by resolved on a 0.8% agarose gel. The restricted DNA fragments were transferred onto a positively charged Nylon membrane (GeneScreen, PerkinElmer). Southern blotting and hybridization with radiolabeled DNA probes were performed as described previously.^[^ ^8^, ^36^ ^]^ The blot was exposed in a Phosphor screen. The signal on the screen was captured by scanning in an OptiQuant Cyclone Plus machine (Perkin Elmer). To measure resection kinetics, the pixel intensity of target bands were quantified and normalized to that of the *TRA1* probe(control). The resulting values were further normalized to that of the uncut sample.

To test the viability of DSB repair by ectopic recombination, cells were cultured in YEP‐Raffinose overnight to the log phase. Cells were then diluted and plated on YEPD or YEP‐Gal plates, followed by incubating at 30 °C for 3 to 5 days. Viability (%) = (the number of colonies grown on YEP‐Gal)/(the number of colonies grown on YEPD x dilution fold) x 100%. At least three independent experiments were performed for each strain. The repair kinetics were monitored by Southern blot as described.^[^ ^42^, ^75^ ^]^ The blot was exposed in a Phosphor screen. The signal on the screen was captured by scanning in an OptiQuant Cyclone Plus machine (Perkin Elmer). The pixel intensity of target bands were quantified and normalized to that of corresponding parental bands on blots. The resulting values were further normalized to that of the control sample (uncut).

### Chromatin Immunoprecipitation (ChIP)

DSB induction, sample processing, and ChIP assay were carried out as previously described.^[^ ^36^ ^]^ Exponentially growing cells (1.2 × 10^7^ cells mL^−1^) in YEP‐Raffinose medium were subject to DSB induction by the addition of 2% galactose. Samples were collected at indicated time points. Cells were lysed on a bead beater, and chromatin DNA was sheared to an average size of ≈300 bp with a Diagenode Bioruptor. Anti‐FLAG antibody was purchased from Cell Signaling Technology. Purified DNA was analyzed by real‐time quantitative PCR with primers that specifically anneal to DNA sequences located at indicated distances from the DSB using the following conditions: 95 °C for 10 min; 40 cycles of 95 °C for 15 s, 60 °C for 1 min, and 72 °C for 30 s.

### Western Blotting

Whole‐cell yeast extracts were prepared using a trichloroacetic acid (TCA) method, as previously described.^[^ ^36^ ^]^ Human cells were lysed in NETN buffer (10 × 10^−3^ m Tris‐HCl, pH 8.0, 100 × 10^−3^ m NaCl, 1 × 10^−3^ m EDTA, and 0.5% NP‐40) with protease inhibitors (Roche) on ice for 30 min. Samples were resolved on an 8% or 12% SDS‐PAGE gel and transferred onto a PVDF membrane (Immobilon‐P; Millipore) using a semi‐dry method. Anti‐HA and anti‐FLAG antibodies were purchased from MBL and Sigma, respectively. Anti‐His and anti‐GST antibodies were obtained from Abclone. GAPDH was purchased from GeneTex. Mouse anti‐TAF1 (6B3), anti‐Myc (9E10) and anti‐p21 (sc‐6246) antibodies were bought from Santa‐Cruz. The antibodies against H4K5ac, H4K8ac, H4K12 or H4K16ac were purchased from Millipore. Anti‐mouse and rabbit IgG HRP‐conjugated secondary antibodies were purchased from Santa Cruz Biotechnology. Blots were developed using the Western Blotting substrate (Bio‐Rad).

### Mass Spectrometry

Yeast cells treated with 0.1% MMS (for 1.5 h) were collected and lysed on a bead beater in lysis buffer (100 × 10^−3^ m HEPES, pH 8.0, 20 × 10^−3^ m MgCl_2_, 150 × 10^−3^ m NaCl, 10% glycerol, 0.4% Nonidet P‐40, 0.1 × 10^−3^ m EDTA plus protease and phosphatase inhibitors). The recombinant GST‐DBD immobilized to the beads was incubated with yeast cell lysates to carried out pull‐down assays. The GST‐DBD associated proteins were eluted using 50 × 10^−3^ m of reduced glutathione (in 50 × 10^−3^ m Tris‐HCl pH 8.0). Eluted proteins were boiling at 95 °C for 5 min. Mass spectrometry analysis followed the procedure previously described by Link et al.^[^ ^76^ ^]^

### Recombination Protein Expression and GST Pull‐Down Assay

Recombinant 6xHis‐ or GST‐ tagged fusion proteins were expressed in E. coli (*BL21*) cells by cloning the coding sequences into the pET30a(+) or pGEX4T3 vector. Protein expression was induced by the addition of 0.1 × 10^−3^ m IPTG at 0.8 OD_600_. Cells were cultured overnight at 16 °C before harvest. After centrifugation at 4000 rpm for 20min, the cell pellets were collected and frozen at ‐80 °C until use.

For GST pull‐down assay, the cell pellet was then resuspended in lysis buffer (20 × 10^−3^ m Tris–HCl, pH 7.4, 50 × 10^−3^ m NaCl, 0. 5 × 10^−3^ m EDTA, 10% glycerol) and lysed by sonication. After centrifugation, the supernatant containing GST or GST fusion proteins were immobilized onto GSH‐sepharose beads (ABclonal) by gently mixing at 4 °C for 1h. After washing with lysis buffer, the resin was then incubated with the His‐tagged proteins or preassembled nucleosomes at 4 °C for 4hrs on a rotator. The beads were washed extensively with wash buffer (20 × 10^−3^ m Tris–HCl, pH 7.4, 200 × 10^−3^ m NaCl, 0. 5 × 10^−3^ m EDTA), and bound proteins were eluted and detected by Western blot or Coomassie brilliant blue staining of SDS‐PAGE.

### Co‐Immunoprecipitation (Co‐IP)

Yeast cells culture (A600 ≈ 1.0) with or without MMS treatment were collected and lysed on a bead beater in lysis buffer (100 × 10^−3^ m HEPES, pH 8.0, 20 × 10^−3^ m MgCl_2_, 150 × 10^−3^ m NaCl, 10% glycerol, 0.4% Nonidet P‐40, 0.1 × 10^−3^ m EDTA plus protease and phosphatase inhibitors). 500U mL^−1^ of benzonase (YEASEN) was added to each sample prior to cell lysis. The extract was clarified by centrifugation at 12,000 g for 10 min at 4 °C, followed by incubating with protein G‐agarose beads for 1 h at 4 °C to preclear non‐specific binding. After centrifugation, the supernatant was incubated with anti‐HA(MBL) or anti‐FLAG (Sigma) antibody at 4 °C overnight with agitation. Protein G‐agarose beads were added, and the mixtures were incubated for another 3 h at 4 °C. Subsequently, the beads were subjected to extensive washing with the lysis buffer at 4 °C. Immunoprecipitated proteins were eluted by boiling beads in a 2xSDS loading buffer for 5 min.

### Isothermal Titration Calorimetry (ITC) Assay

For ITC measurement, the acetylated or unacetylated H4 peptide (1–22 aa) was commercially synthesized at GenScript. Recombinant 6xHis‐tagged fusion proteins (DBD, 2YF‐DBD, TAF1‐DBD) were purified by incubation with the nickel sepharose resin followed by an extensive wash. The purified proteins were dialyzed against the ITC buffer (100 × 10^−3^ m NaCl, 50 × 10^−3^ m Tris‐HCl, pH 7.5). During the ITC assay, the fusion proteins (≈50 × 10^−6^ m for DBD or 2YF‐DBD, ≈20 × 10^−6^ m for TAF1‐DBD) were titrated with each peptide (≈1 × 10^−3^ m for DBD and 2YF‐DBD or 0.5 × 10^−3^ m for TAF1‐DBD) separately at 25 °C. Each ITC titration comprised 19 successive injections. The resultant ITC curves were processed using Origin (8.0) software in accordance with the “‘One Set of Sites”’ fitting model.

### Human DNA Repair Reporter Assay

HR or NHEJ reporter assay was performed with the U2OS cells integrated with DR‐GFP or EJ5‐GFP cassettes, as previously reported.^[^ ^56^ ^]^ Cells transfected with indicated plasmids were then transiently transfected with I‐*Sce*I expression vector pCBAScel (Addgene). 48hr later, the percentage of GFP positive cells was analyzed by flow cytometry. The HR or NHEJ efficiency was presented as a percentage of control cells. The values presented are the analysis of three independent experiments. At least 10 000 cells were counted for each sample. The statistical data were from three independent experiments.

### Cell Cycle Analysis

HCT116 cells transfected with indicated plasmids were collected during the exponential growth phase and were fixed in 70% ethanol at 4 °C overnight. Fixed cells were treated with RNAse A (100 µg mL^−1^) (Omega) and propidium iodide (200 µg mL^−1^) (Sigma) for 30 min at 37 °C. Data were acquired using the BD celesta flow cytometry and analyzed with flow jo software. Data were presented as the mean of three independent experiments.

### Statistical Analysis

All experiments were performed in at least three biological replicates, and no data were excluded from the analysis. For ChIP experiments, the signal was normalized to that of the “0” time point (before DSB induction) or the control sample (IgG). For comparison of two groups, data analysis was performed by Students’ t test and data were presented as mean ± SD. For comparison of multiple groups (>2), data analysis was performed by one‐way ANOVA followed by Turkey post‐hoc test or two‐way ANOVA, and data were presented as mean ± SEM. All statistics were carried out with either Microsoft Excel or GraphPad Prism 9. Significance was considered as *p < 0.05; ** *p* < 0.01, *** *p* < 0.001.

## Conflict of Interest

The authors declare no conflict of interest.

## Author Contributions

H.P., S.Z., and Y.P. contributed equally to this work. H.Y.P. and S.Z. conducted the majority of yeast work. Y.P. performed the human in vivo experiments. S.Z., S.Y.Z, and X.Z. constructed some plasmids, yeast strains, and performed the drug sensitivity screen. Q.L., X.H.Z., S.Y., G.L., Y.D., X.G, and X.C.Z. guided and participated in protein expression and purification. H.Y.P., S.Z., and Y.P. designed most of the experiments. X.C. and H.D.P. supervised the work and wrote the manuscript.

## Supporting information

Supporting Information

## Data Availability

The data that support the findings of this study are available from the corresponding author upon reasonable request.
