# Supplementary material for: Yeast Bromodomain Factor 1 and Its Human Homolog TAF1 Play Conserved Roles in Promoting Homologous Recombination
Source: Adv Sci (Weinh). 2021 May 30;8(15):2100753. doi: 10.1002/advs.202100753 (PMC8336524; doi:10.1002/advs.202100753)
Supplement: Supplementary file 1 — Supporting Information [file ADVS-8-2100753-s001.pdf]

© 2021 Wiley-VCH GmbH

ADVANCED SCIENCE
Open Access

Supporting Information

for *Adv. Sci.*, DOI: 10.1002/advs.202100753

Yeast Bromodomain Factor 1 and Its Human Homolog TAF1 Play Conserved Roles in Promoting Homologous Recombination

Haoyang Peng[1,4], Simin Zhang[1,4], Yihan Peng[2,4], Shuangyi Zhu[1], Xin Zhao[1], Xiaocong Zhao[1], Shuangshuang Yang[3], Guangxue Liu[1], Yang Dong[1], Xiaoli Gan[1], Qing Li[3], Xinhua Zhang[1], Huadong Pei[2,], Xuefeng Chen[1,*]*

Figure S1

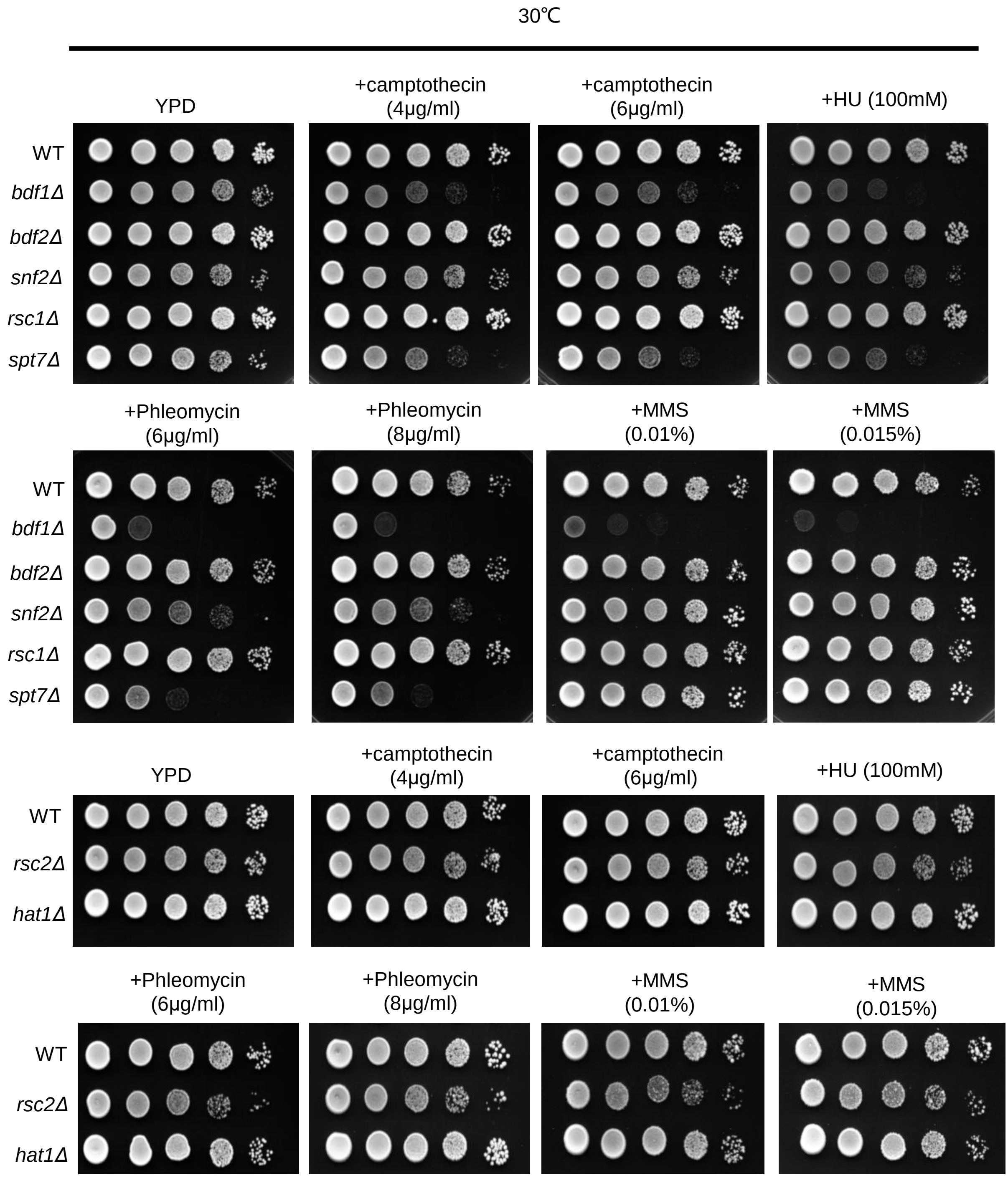
30℃
YPD
+camptothecin (4μg/ml)
+camptothecin (6μg/ml)
+HU (100mM)
WT
bdf1Δ
bdf2Δ
snf2Δ
rsc1Δ
spt7Δ
+Phleomycin (6μg/ml)
+Phleomycin (8μg/ml)
+MMS (0.01%)
+MMS (0.015%)
WT
bdf1Δ
bdf2Δ
snf2Δ
rsc1Δ
spt7Δ
YPD
+camptothecin (4μg/ml)
+camptothecin (6μg/ml)
+HU (100mM)
WT
rsc2Δ
hat1Δ
+Phleomycin (6μg/ml)
+Phleomycin (8μg/ml)
+MMS (0.01%)
+MMS (0.015%)
WT
rsc2Δ
hat1Δ

Figure S1 continued

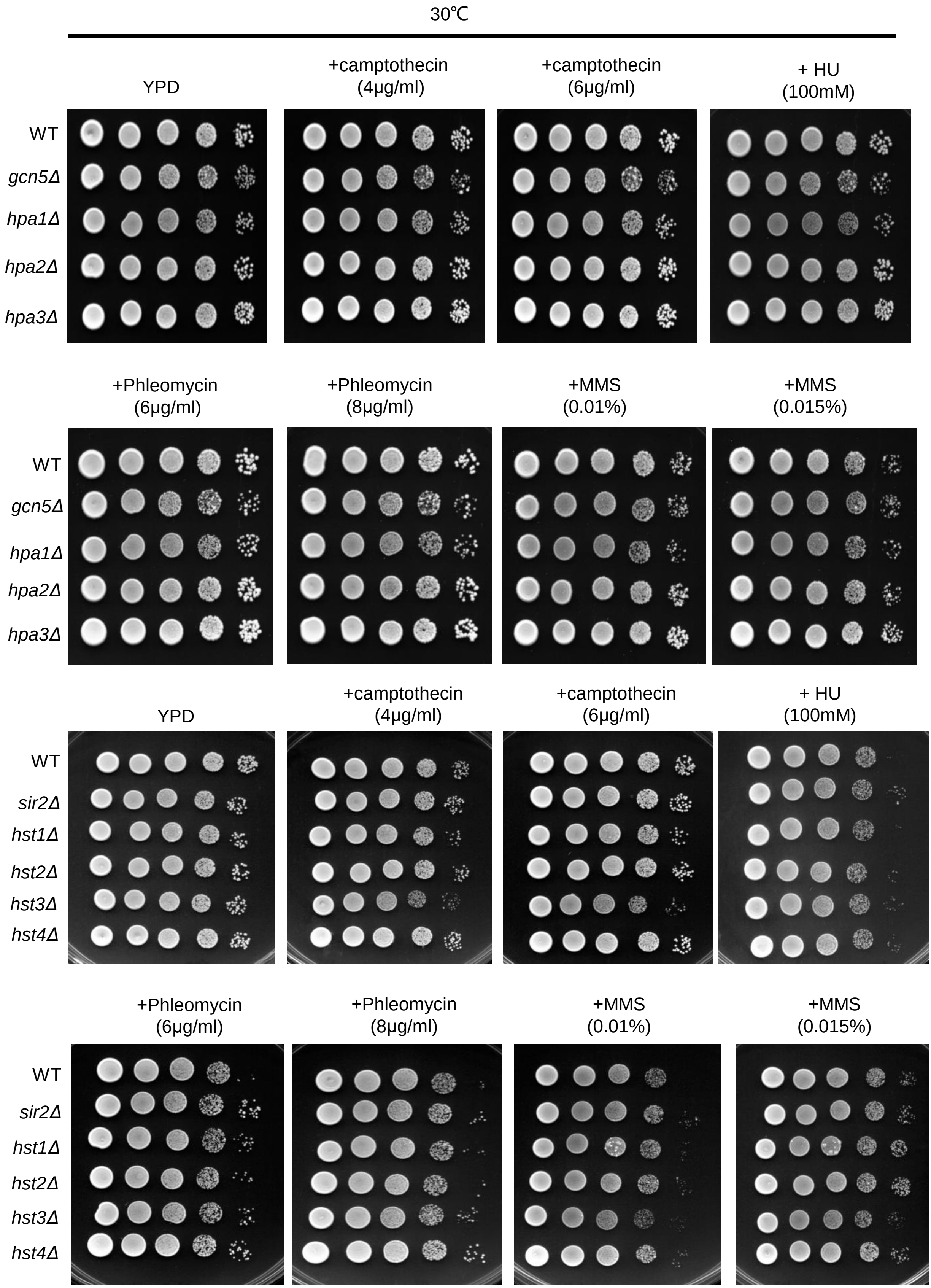

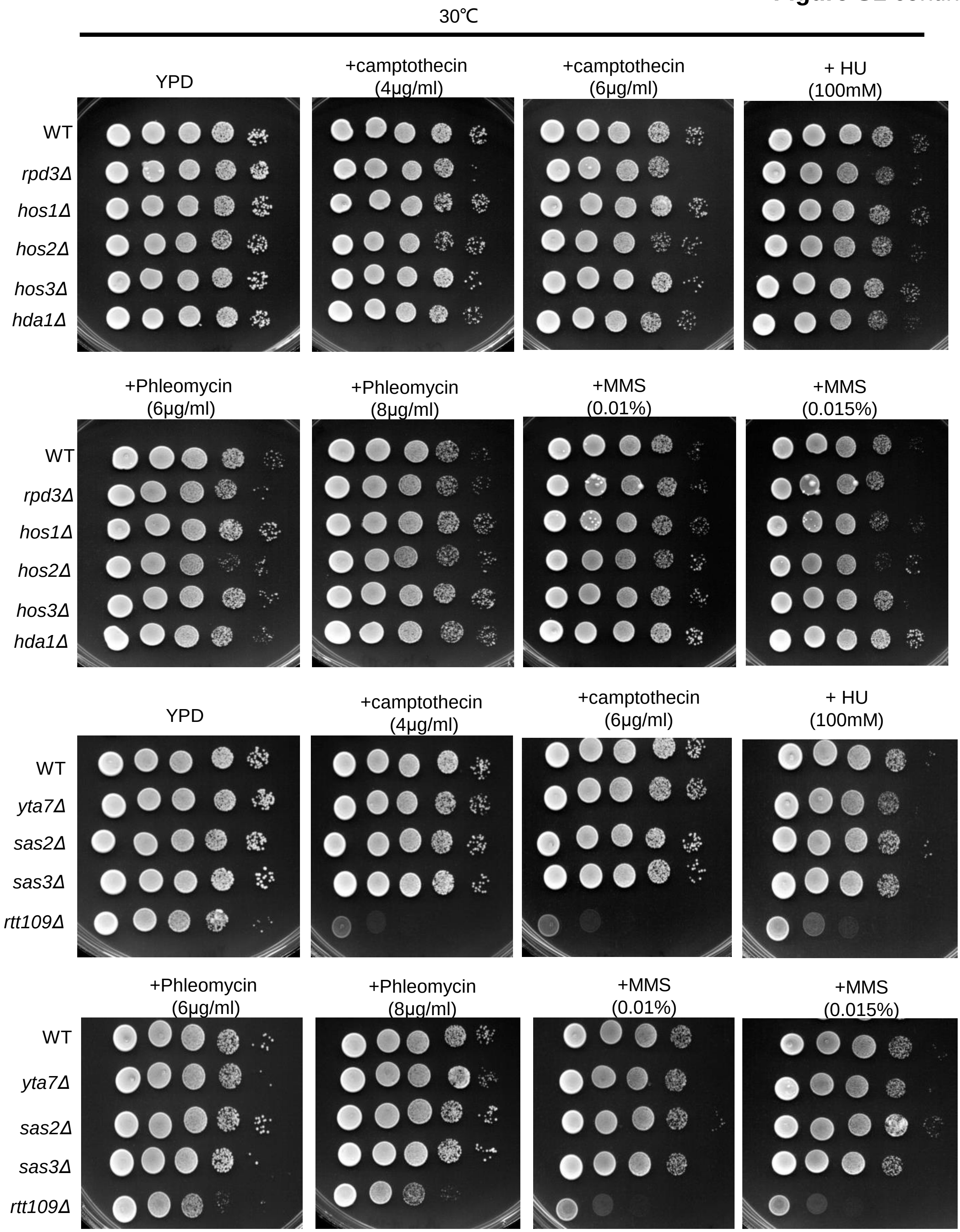

Figure S1 Screen for acetylation-related yeast mutants with enhanced DNA damage sensitivity. The WT and mutant strains were taken from the yeast knockout collection in BY4741 background. 10-fold serial dilutions of indicated cultures on plates with camptothecin, HU, phleomycin, or MMS at indicated concentrations. Plates were incubated at 30° C for 3-4 days.

Figure S2

A

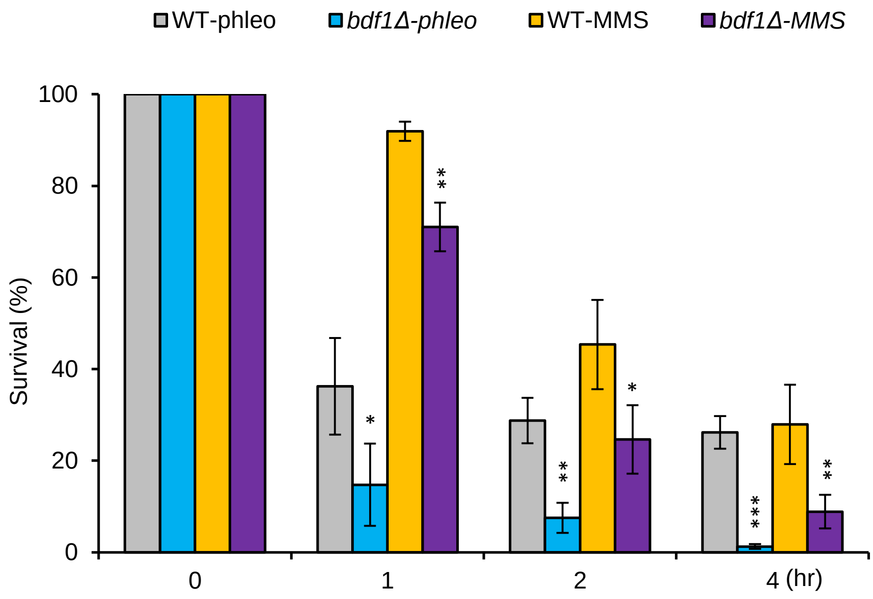

B

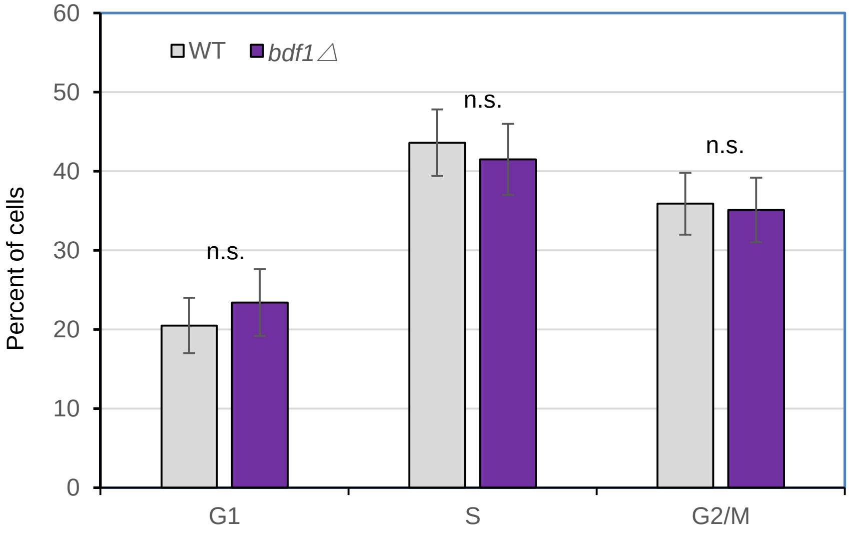

Figure S2 The deletion of *BDF1* reduces the cell survival upon DNA damage but does not alter the cell cycle distribution. **A**, Survival rate for the WT or *bdf1*△ mutant cells upon phleomycin (10μg/ml) or MMS (0.1%) treatment (n=3). **B**, Plot showing the distribution of unperturbed WT or *bdf1*△ cells in each cell cycle stage (n=3). Data are presented as mean ± S.D., *p*-values are calculated using unpaired two-tailed Student's t-test, **p*<0.05, ***p*<0.01, ****p*<0.001, n.s.: no significance.

Figure S3

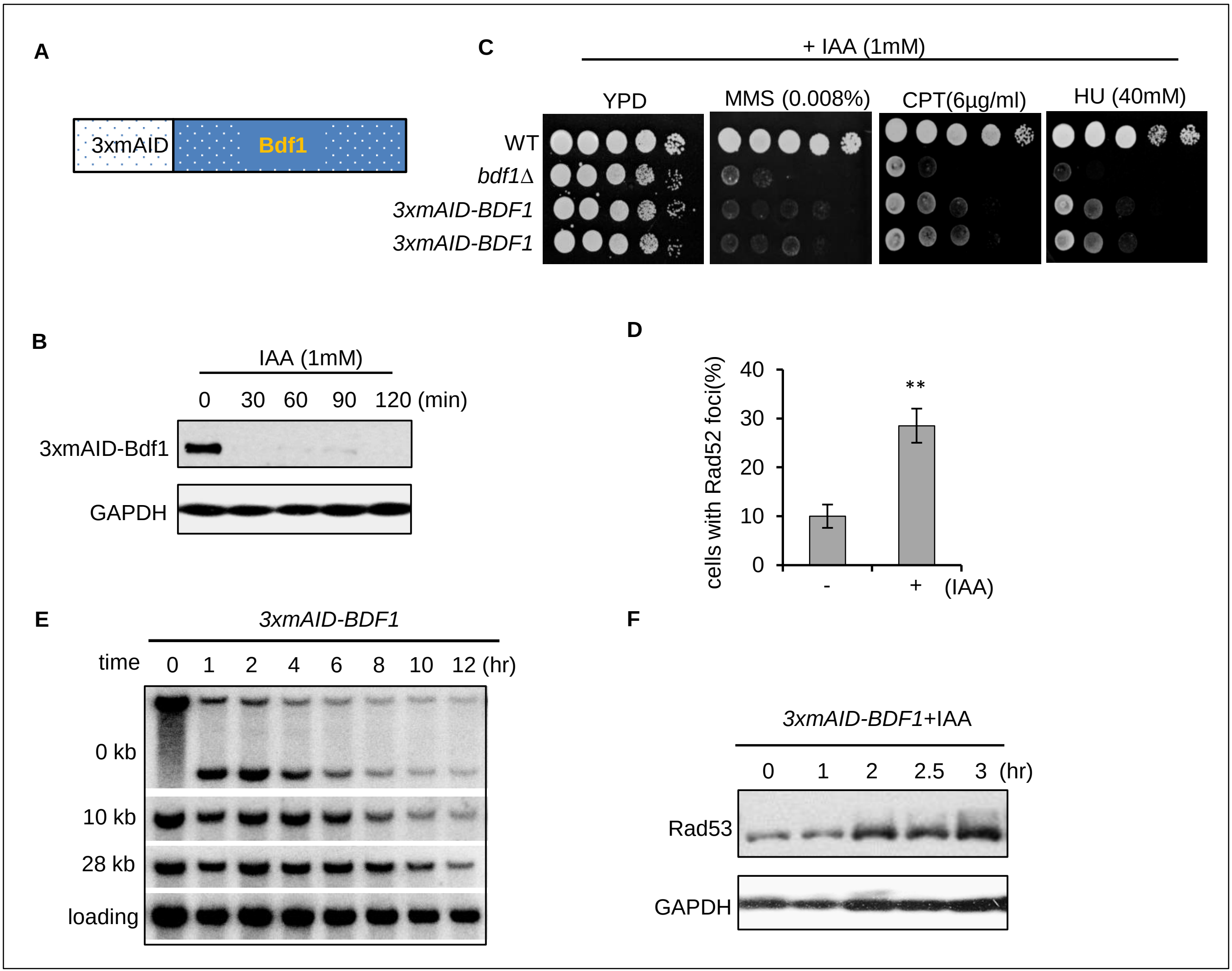

Figure S3 The depletion of Bdf1 by the AID-degron impairs DNA damage response. A, Scheme showing the Bdf1 fusion protein with a 3xmAID tag at the N-terminus. **B**, Western blot monitoring the degradation of 3xmAID-Bdf1 upon the addition of IAA (1mM). GAPDH served as a loading control. **C**, DNA damage sensitivity test for indicated cells at indicated drug concentrations in the presence of IAA. **D**, The ratio of spontaneous Rad52-YFP foci in *3xmAID-BDF1* cells before or after the addition of IAA. Data are presented as mean ± S.D., p-values are calculated using unpaired two-tailed Student's t-test, ***p*<0.01. **E**, Southern blot analysis of resection kinetics for *3xmAID-Bdf1* cells upon IAA treatment. The quantification data is shown in Figure 1F. **F**, Western blot showing Rad53 phosphorylation in *3xmAID-BDF1* cells at different time points after IAA addition.

Figure S4

A (*bdf1*△/ WT)

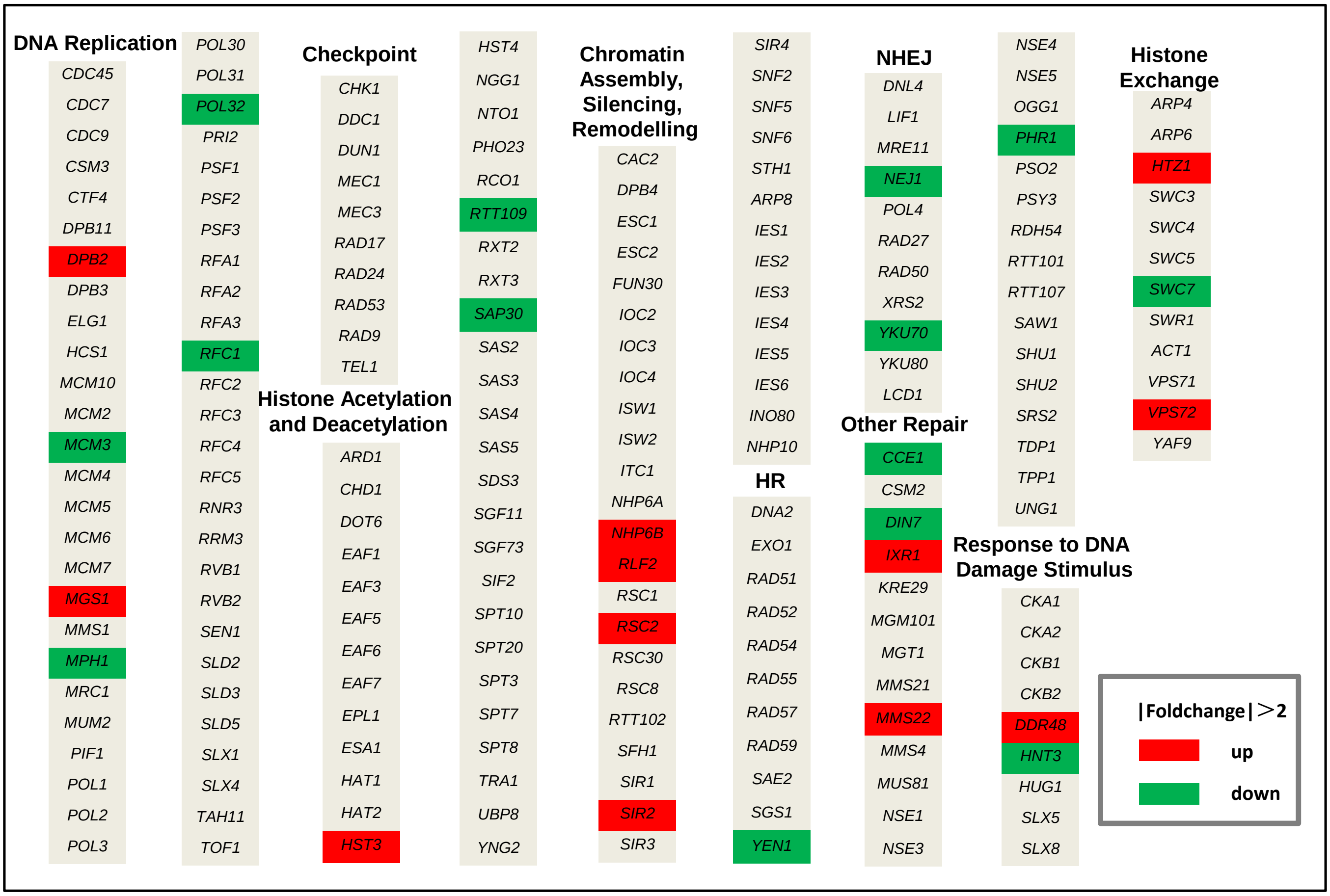

B **Figure S4** continued

(*bdf1-2YF* / WT)

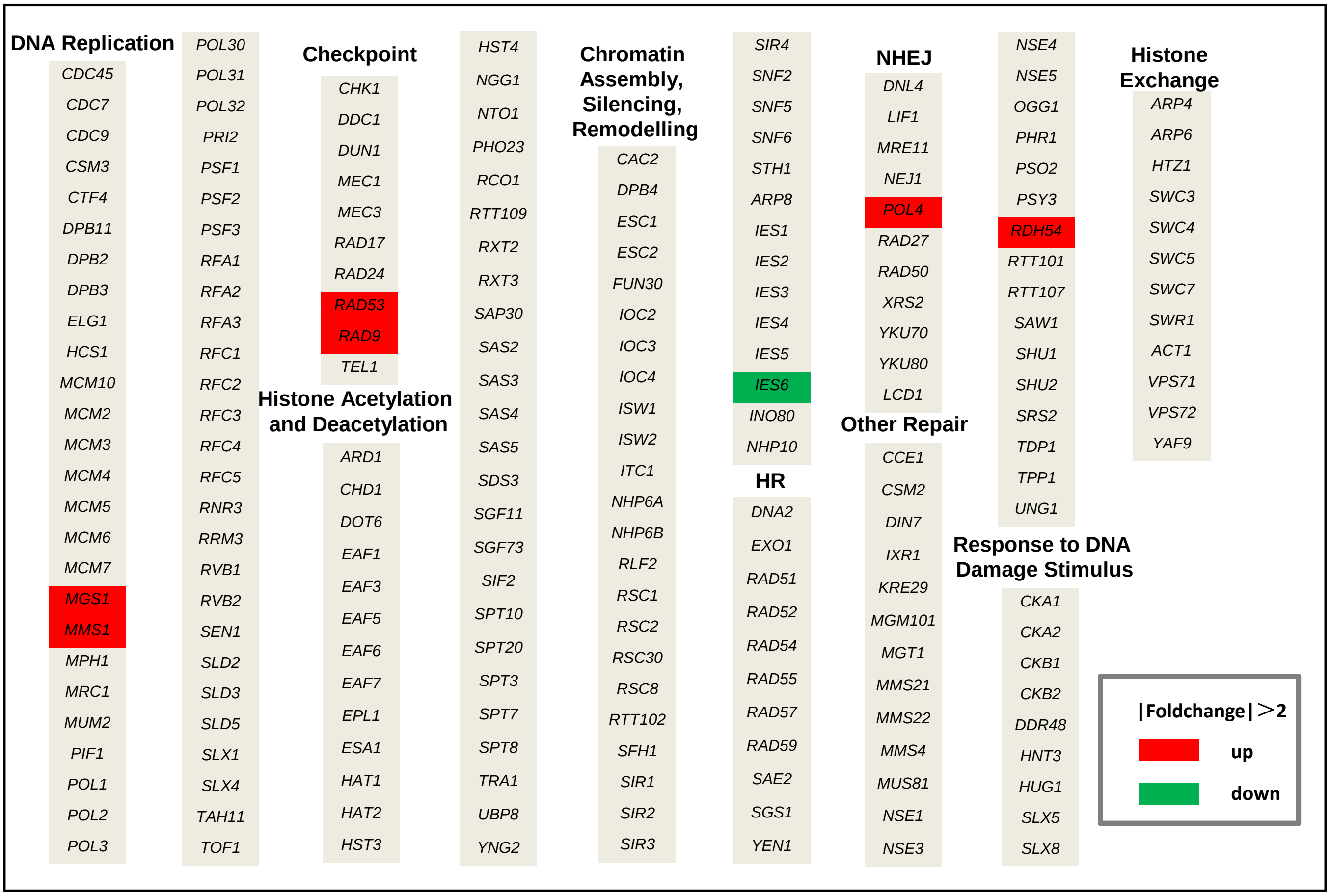

Figure S4 The relative expression levels of the DNA damage response and repair genes in the WT, *bdf1Δ* or *bdf1-2YF* mutant. A. Relative expression of the DNA damage response and repair genes between the WT and *bdf1Δ* mutant cells. B. Relative expression of the DNA damage response and repair genes between the WT and *bdf1-2YF* mutant cells. The results were obtained from the RNA-seq data. The genes with altered expression (> 2 folds or <0.5 fold) in the mutant cells are marked in red or green color, as indicated.

Figure S5

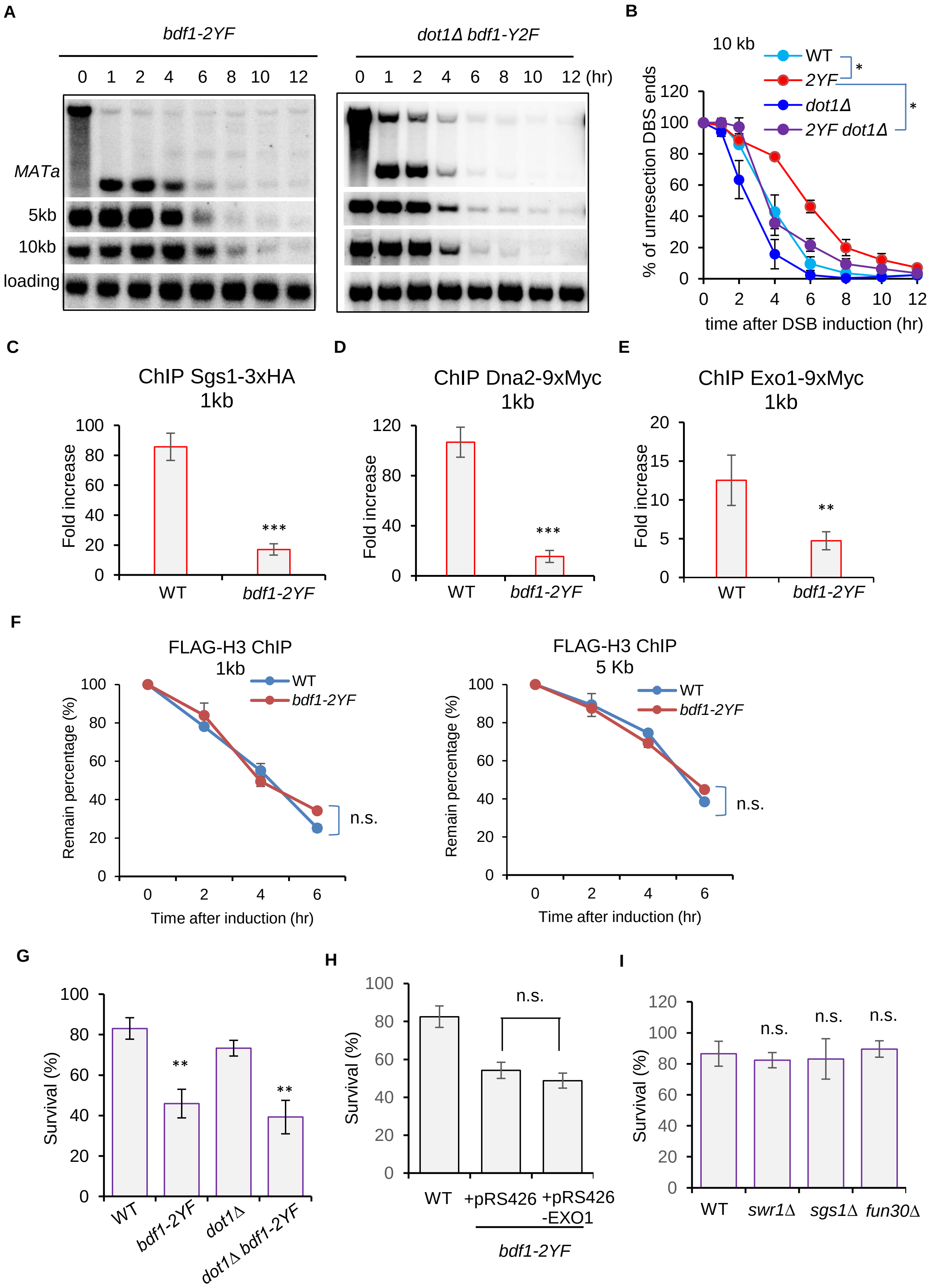
A
bdf1-2YF
dot1Δ bdf1-Y2F
0 1 2 4 6 8 10 12
0 1 2 4 6 8 10 12 (hr)
MATa
5kb
10kb
loading
B
10 kb
WT
2YF
dot1Δ
2YF dot1Δ
*
*
% of unresection DBS ends
time after DSB induction (hr)
C
ChIP Sgs1-3xHA 1kb
Fold increase
***
WT
bdf1-2YF
D
ChIP Dna2-9xMyc 1kb
Fold increase
***
WT
bdf1-2YF
E
ChIP Exo1-9xMyc 1kb
Fold increase
**
WT
bdf1-2YF
F
FLAG-H3 ChIP 1kb
WT
bdf1-2YF
Remain percentage (%)
n.s.
Time after induction (hr)
FLAG-H3 ChIP 5 Kb
WT
bdf1-2YF
Remain percentage (%)
n.s.
Time after induction (hr)
G
Survival (%)
**
**
WT
bdf1-2YF
dot1Δ
dot1Δ bdf1-2YF
H
Survival (%)
n.s.
WT
+pRS426
+pRS426 -EXO1
bdf1-2YF
I
Survival (%)
n.s.
n.s.
n.s.
WT
swr1Δ
sgs1Δ
fun30Δ

Figure S5 The deletion of *DOT1* or overexpression of *EXO1* failed to rescue the HR defect in *bdf1-2YF* cells. **A-B**, Southern blot analysis and quantification of resection kinetics for indicated cells. Data analysis was performed by two-way ANOVA and data were presented as mean ± SEM (n=3). *p<0.05. **C-E**, ChIP-qPCR showing the enrichment of Sgs1-3xHA (C), Dna2-9xMyc (D) and Exo1-9xMyc (E), respectively, in the WT and *bdf1-2YF* cells. Samples were collected at 4hrs post galactose induction. The ChIP signal was normalized to that of the "0" time point sample (before DSB induction). Data are presented as mean ± S.D., n=3, p-values are calculated using unpaired two-tailed Student's t-test, **p<0.01, ***p<0.001. **F**, ChIP showing H3 loss at 1kb or 5 kb upstream of the DSB in WT or *bdf1-2YF* mutant. Fold changes was calculated by normalizing the ChIP signals to that of the "0" time point (before DSB induction). Data analysis was performed by two-way ANOVA and data were presented as mean ± SEM. n=3. n.s.: no significance. **G-H.** Survival rate of ectopic recombination for indicated strains. Data are presented as mean ± S.D., n=3, p-values are calculated using unpaired two-tailed Student's t-test, **p<0.01, n.s.: no significance. **I**. Survival rate of ectopic recombination for indicated strains. Data analysis was performed by one-way ANOVA followed by Turkey post-hoc test and data were presented as mean ± SEM. n=3. n.s.: no significance

Figure S6

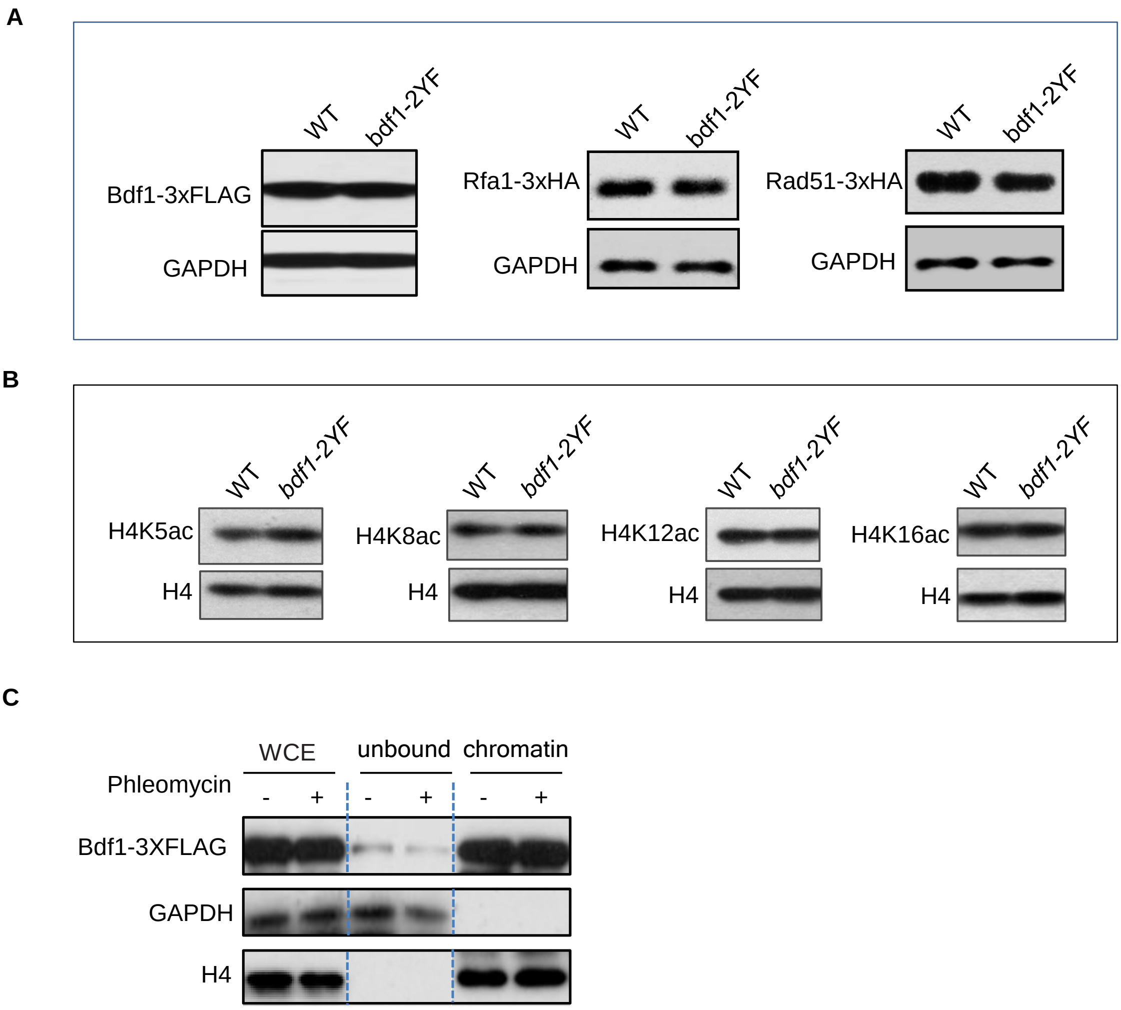

Figure S6 Bdf1 associates with chromatin. A, Western blot indicating the levels of Bdf1-3xFLAG, Rfa1-3xHA, and Rad51-3xHA in the WT or *bdf1-2YF* cells. GAPDH served as the loading control. **B**, Western blot showing the levels of H4K5ac, H4K8ac, H4K12ac, or H4K16ac in the WT or *bdf1-2YF* cells. H4 was used as a loading control. **C**, Chromatin fraction assay showing the subcellular distribution of Bdf1 in the absence or presence of phleomycin treatment (20 µg/ml, 2hr).

Figure S7

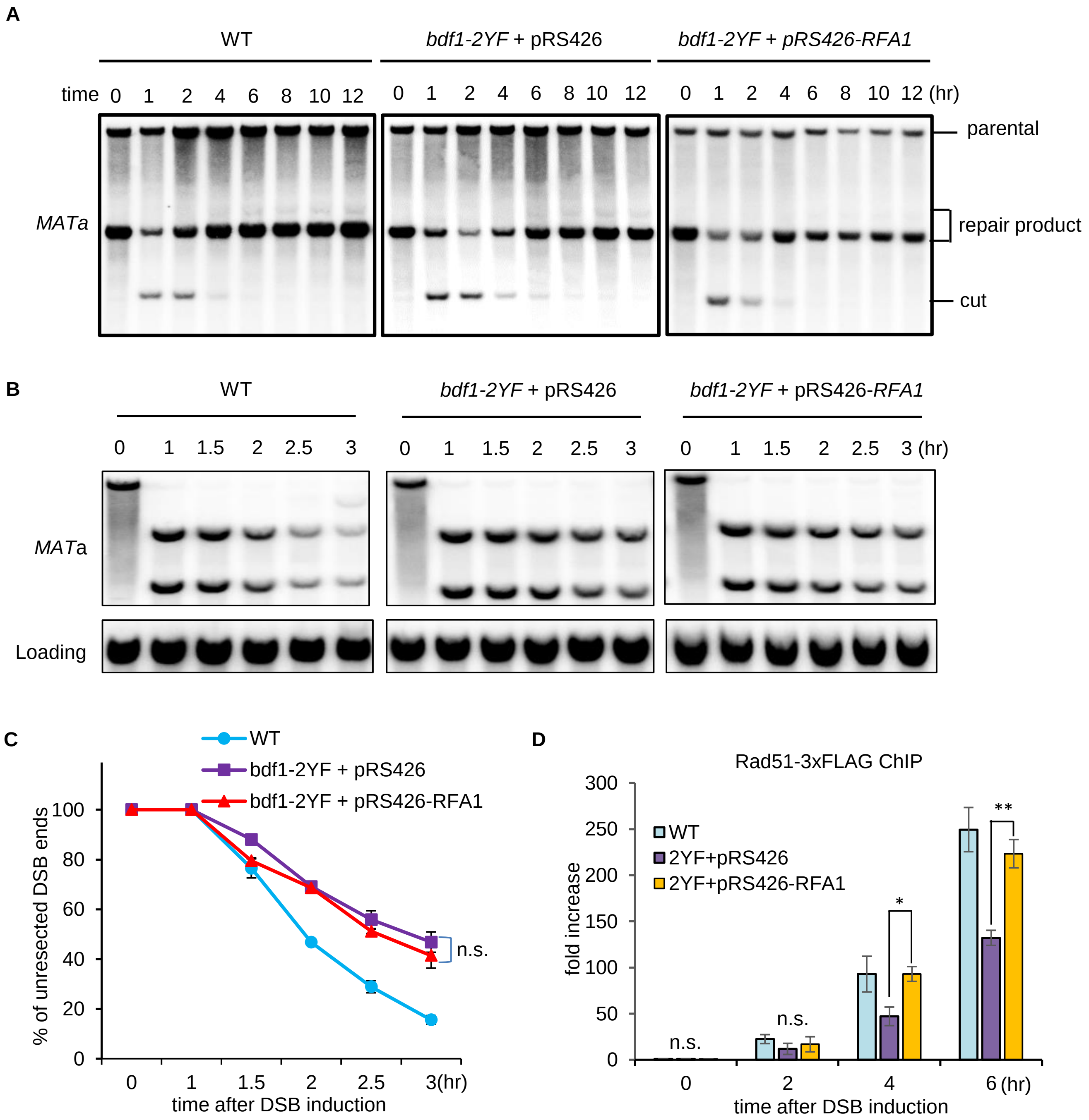

Figure S7 Overexpression of RPA in *bdf1-2YF* cells rescued the defect in HR repair and Rad51 loading. **A**, Southern blot analysis of repair kinetics for ectopic recombination in the WT or *bdf1-2YF* mutant cells complemented with the pRS426 or pRS426-*RFA1* plasmid. The quantification data is presented in Fig. 4H. **B** and **C**, Southern blot analysis and quantification of resection kinetics for the WT or *bdf1-2YF* mutant cells complemented with the pRS426 or pRS426-*RFA1* plasmid*.* The *TRA1* probe was used as a loading control. Data analysis was performed by two-way ANOVA and data were presented as mean ± SEM. n=3. **D**, ChIP-qPCR showing the loading of Rad51-3xFLAG at DSBs in the WT or *bdf1-2YF* cells harboring the pRS426 or pRS426-*RFA1* plasmid. Data are presented as mean ± S.D., n=3, *p*-values are calculated using unpaired two-tailed Student's *t*-test, **p*<0.05, ***p*<0.01. n.s.: no significance.

Figure S8

A

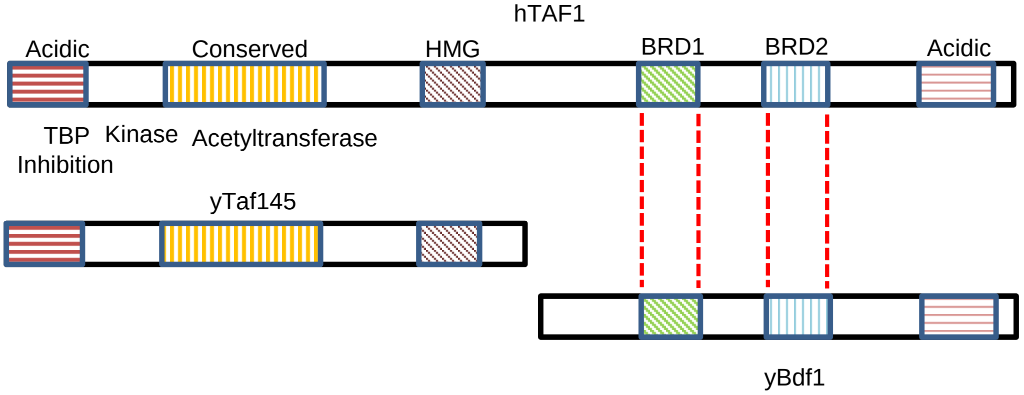

B

(Identity: 26.9%; similarity: 45.3%; Gap: 23.2%)

```
hTAF1   1297 EEQEEELEKTVIHNDNEELIKVEGTKIVLGKQLIESADEVRRKSLVLKFPKQ--QLPPKKKRRVGTTVHCDYLNRPHKSI--HRRRTDPMVTLSSILESI  1392
             |::::.|:|        ||  ..:|||        .|..||..::.|.::.||:  ..||.:.          |..|.|...|  |:::       .::|  .
yBdf1     95 EDEQQGLKK-------EE--GGQGTK-------QEDLDENSKQELPMEVPKEPAPAPPPEP---------DMNNLPQNPIPKHQQK-------HALL--A   160

hTAF1   1393 INDMRDLPNTYPFHTPVN-AKV-VKDYYKIITRPMDLQTLRENVRKRLYPSREEFREHLELIVKNSATYNGPKHSLTQISQS--------MLDLCDEK--  1480  (Y1417)
             |..::.|.:..||..||: .|: :..|:..|.|||||.|:...:....|...|:..|...|:|.||..:|||...::|::::        ||::..:.
yBdf1    161 IKAVKRLKDARPFLQPVDPVKLDIPFYFNYIKRPMDLSTIERKLNVGAYEVPEQITEDFNLMVNNSIKFNGPNAGISQMARNIQASFEKHMLNMPAKDAP  260  (Y187)

hTAF1   1481 ------------------LKEKEDKLARLEKAINPLLDDDD-------------QVAFSF---ILDNIVTQKMMAVPDSWPFHHPVN--KKFVPDYYKVI  1544  (Y1540)
                               ::..:....|.::.|:|....|.              |.|..|   :|..::.:|..:.  ::||..||:  ...:|.|:..:
yBdf1    261 PVIAKGRRSSAQEDAPIVIRRAQTHNGRPKRTIHPPKSKDIYPYESKKPKSKRLQQAMKFCQSVLKELMAKKHASY--NYPFLEPVDPVSMNLPTYFDYV  358  (Y354)

hTAF1   1545 VNPMDLETIRKNISKHKYQSRESFLDDVNLILANSVKYNGP------------------------------------------ESQYTKTAQE---IVN  1598
             ..||||.||.|.::..:||:.|.|..||.|:..|...:|..                                          ||:|:::..:   |.|
yBdf1    359 KEPMDLGTIAKKLNDWQYQTMEDFERDVRLVFKNCYTFNPDGTIVNMMGHRLEEVFNSKWADRPNLDDYDSDEDSRTQGDYDDYESEYSESDIDETIITN  458
```

Figure S8 Bdf1 is homologous to the C-terminal half of human TAF1. A, The relationship between yeast Bdf1, Taf145 and human TAF1. Yeast Taf145 corresponds to the N-terminal part of human TAF1, while Bdf1 corresponds to the C-terminal half of human TAF1. The conserved motifs are indicated. **B**, Alignment of peptide sequences for the BRDs of yeast Bdf1 and human TAF1. Arrows indicate the tyrosine critical for maintaining the BRD structure.

Figure S9

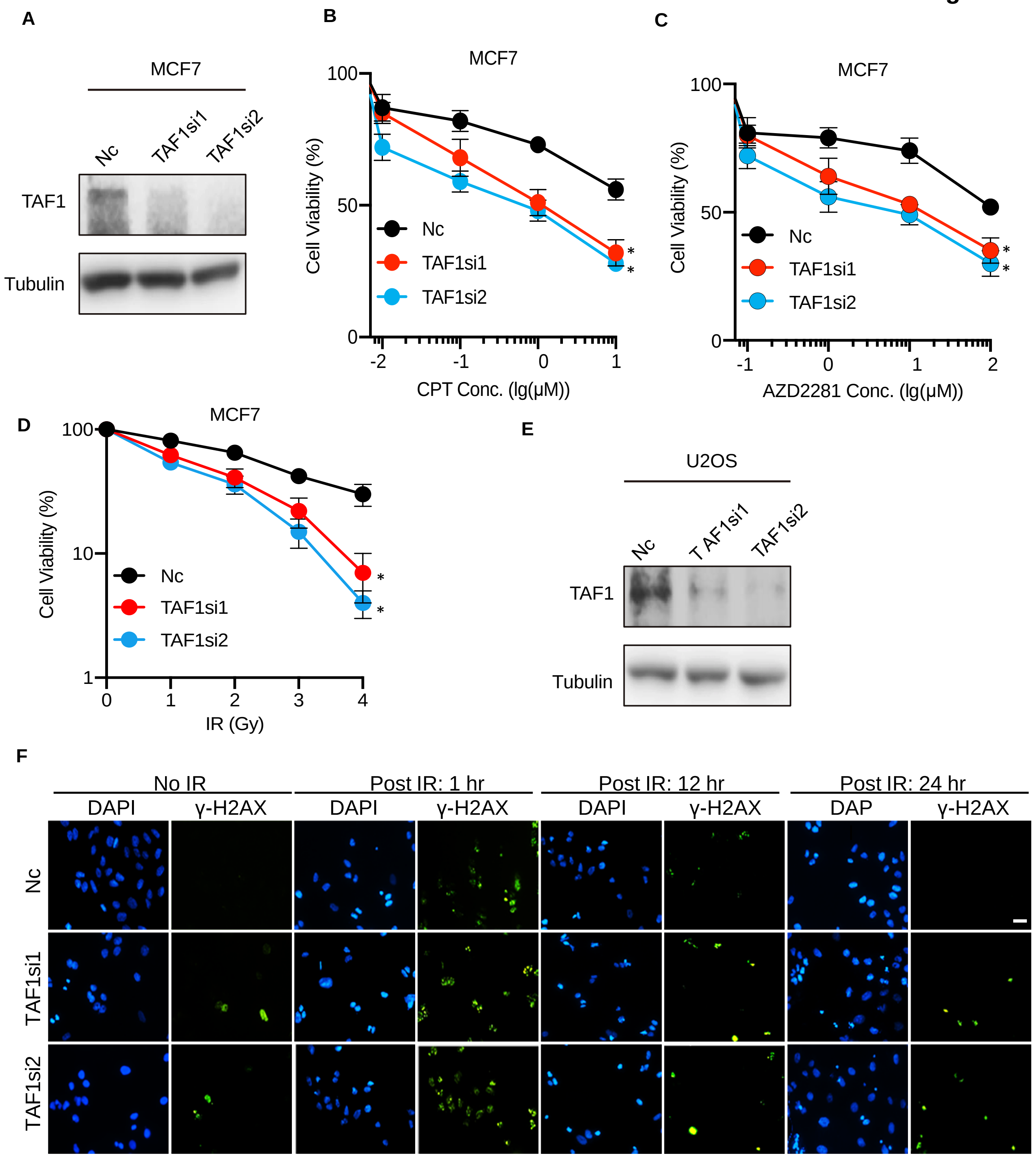

Figure S9 Human TAF1 promotes DNA damage resistance and repair. A and **E**, Western blot showing the protein levels of TAF1 in indicated MCF7 or U2OS cells. **B-D**, Survival curve for the control or TAF1-depleted MCF7 cells upon exposure to CPT, AZD2281 or IR treatment. Data were analyzed by two-way ANOVA and were presented as mean ± SEM; n=3; **p* < 0.05. **F**, Immunostaining of γ-H2AX foci formation during the recovery from IR treatment in the control or TAF1-depleted U2OS cells. The quantification result is presented in Fig 6A. Scale bar: 200 μm.

Figure S10

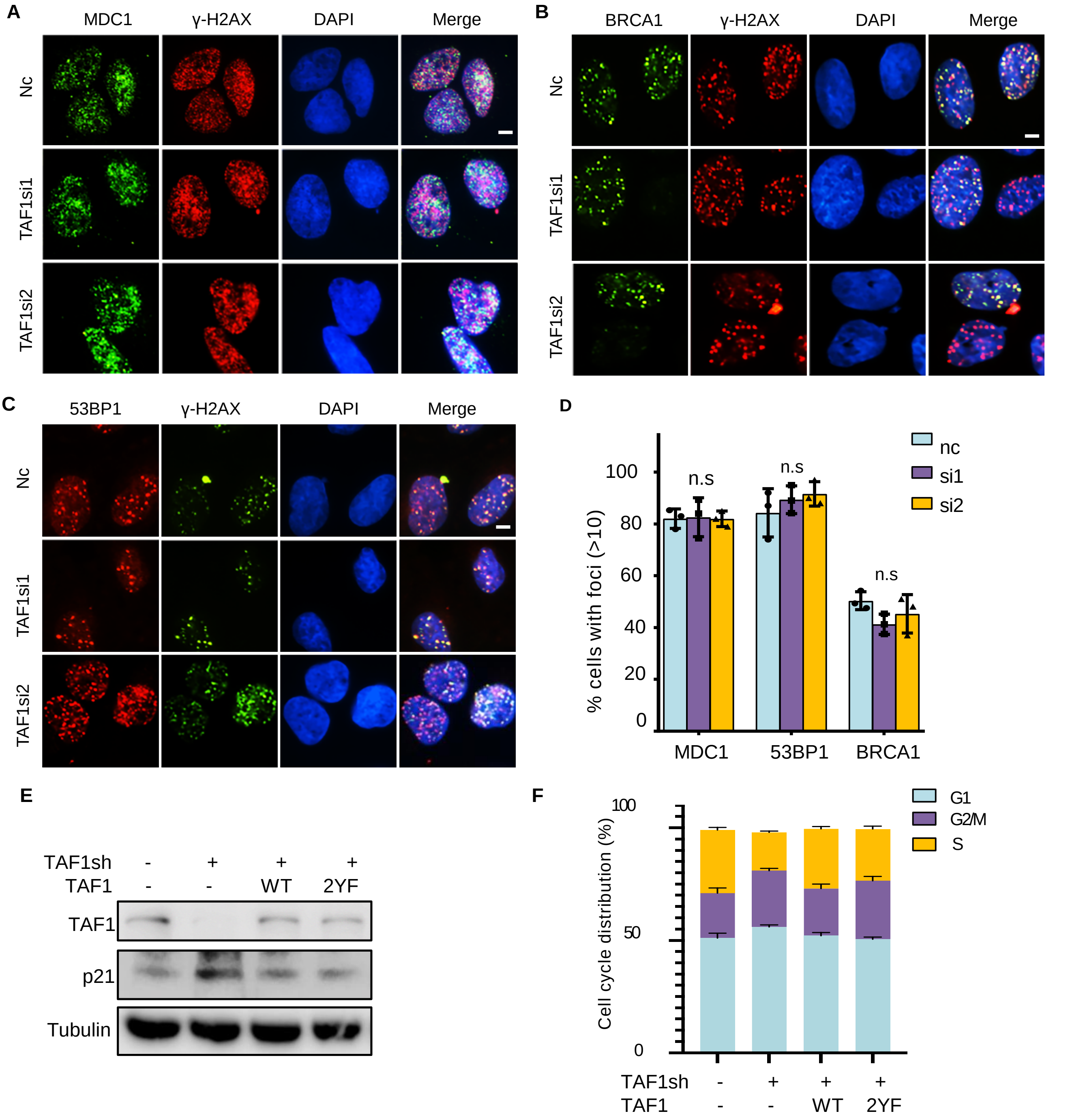

Figure S10 TAF1 depletion does not affect the recruitment of MDC1, BRCA1 and 53BP1. A-C, Immunofluorescent analysis of DNA damage-induced recruitment of MDC1, 53BP1 or BRCA1 in the WT or TAF1 knockdown cells. γ-H2AX foci mark the sites of DNA lesions. Scale bar: 10 μm. **D**, Quantification of A-C are average of three independent experiments, ~100 cells were counted for each experiment. Data were analyzed by Students' *t*-test and were presented as mean ± SD. n.s.: no significance. **E**, Western blot analysis of p21 protein levels in indicated cells. Untreated cells, TAF1 knockdown cells, or TAF1 knockdown cells complemented with a plasmid harboring a WT *TAF1* or a *taf1-2YF* mutant allele. **F**, Plot showing the cell cycle distribution for WT, TAF1 knockdown HCT116 cells, or TAF1 knockdown HCT116 cells complemented with a plasmid harboring a WT *TAF1* or *taf1-2YF* mutant allele. Data were acquired using the BD celesta flow cytometry and analyzed with flow jo software. Data were presented as mean ± SD (n=3).

Figure S11

A

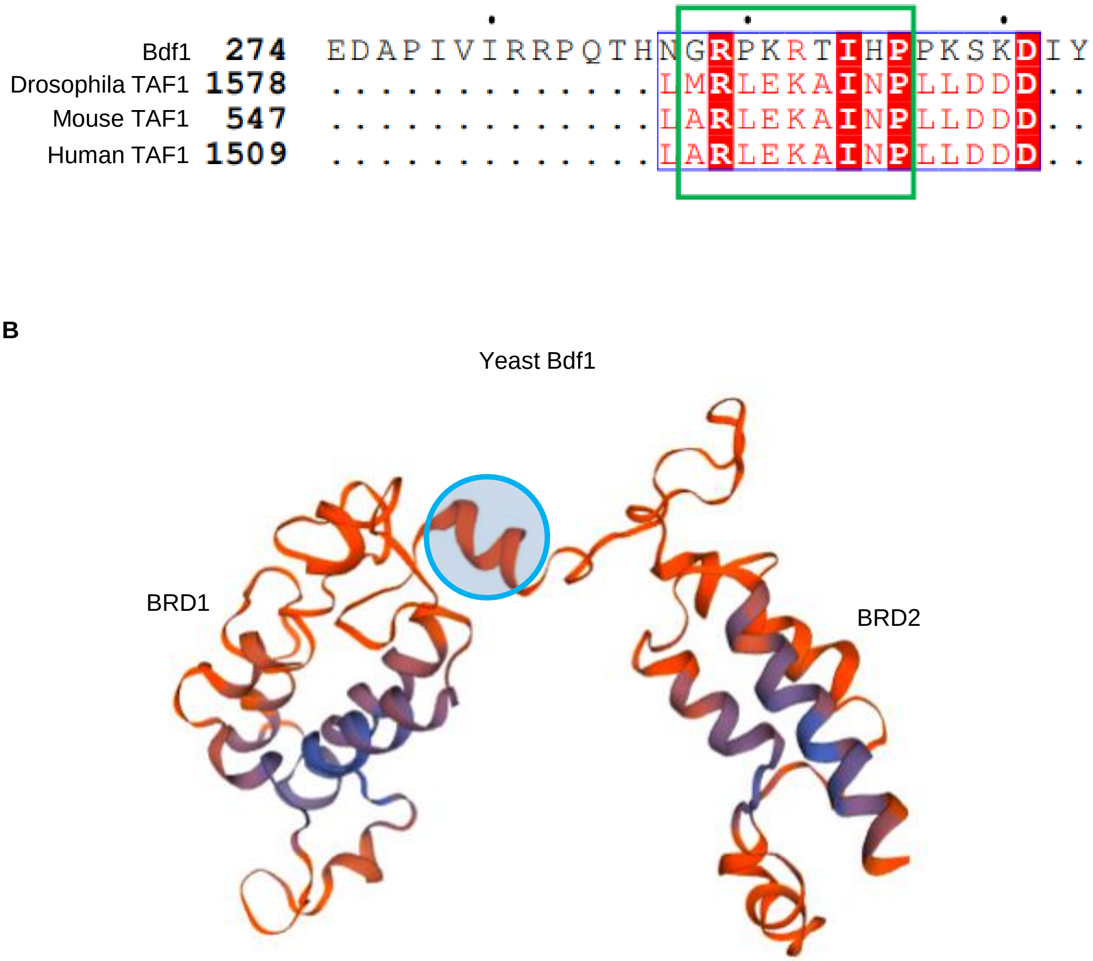

Figure S11. Structure of the BRDs of yeast Bdf1. (**A**) Sequence alignment showing conserved residues in the RPA-interacting motif in Bdf1. (**B**) Structure model of the BRD motifs of Bdf1 (Adapted from the structure of human TAF1, Filippakopoulos et al, Cell, 2012; PDB: template:3uv5.1.A). The RPA-interacting motif in the linker region is marked by the circle.

Supplemental Table 1. Yeast strains

Strain name	Parental name	Genotype	Source
BY4741		*MATa his3Δ1 leu2 met15Δ ura3-5*	
JKM139		*MATa ho hml::ADE1 hmr::ADE1 ade1-100 leu2-3,112 trp1::hisG lys5 ura3-52 ade3::GAL::HO*	1
JKM179		*MATα ade1 leu2-3,112 lys5 trp1::hisG ura3-52 hml::ADE1 hmr::ADE1 ade3::GAL::HO*	1
tGI354		*MATa-inc arg5,6::MATa-HPH ade3::GAL::HO hmr::ADE1 hml::ADE1 ura3-52*	2
BY4732-YAC		*MATα his3Δ200 ura3Δ0 met15Δ0 trp1Δ63/YAC(MFA1pr-HIS3 URA3 MET15 TRP1)*	3
BY4741 hat1Δ	BY4741	*hat1::KanMX*	Open Biosystems
BY4741 gcn5Δ	BY4741	*gcn5::KanMX*	Open Biosystems
BY4741 hpa2Δ	BY4741	*hpa2::KanMX*	Open Biosystems
BY4741 hpa3Δ	BY4741	*hpa3::KanMX*	Open Biosystems
BY4741 sas2Δ	BY4741	*sas2::KanMX*	Open Biosystems
BY4741 sas3Δ	BY4741	*sas3::KanMX*	Open Biosystems
BY4741 rtt109Δ	BY4741	*rtt109::KanMX*	Open Biosystems
BY4741 rpd3Δ	BY4741	*rpd3::KanMX*	Open Biosystems
BY4741 hos1Δ	BY4741	*hos1::KanMX*	Open Biosystems
BY4741 hos2Δ	BY4741	*hos2::KanMX*	Open Biosystems
BY4741 hos3Δ	BY4741	*hos3::KanMX*	Open Biosystems
BY4741 hda1Δ	BY4741	*hda1::KanMX*	Open Biosystems
BY4741 sir2Δ	BY4741	*sir2::KanMX*	Open Biosystems
BY4741 hst1Δ	BY4741	*hst1::KanMX*	Open Biosystems
BY4741 hst2Δ	BY4741	*hst2::KanMX*	Open Biosystems
BY4741 hst3Δ	BY4741	*hst3::KanMX*	Open Biosystems
BY4741 hst4Δ	BY4741	*hst4::KanMX*	Open Biosystems
BY4741 bdf1Δ	BY4741	*bdf1::KanMX*	Open Biosystems
BY4741 bdf2Δ	BY4741	*bdf2::KanMX*	Open Biosystems
BY4741 rsc1Δ	BY4741	*rsc1::KanMX*	Open Biosystems
BY4741 rsc2Δ	BY4741	*rsc2::KanMX*	Open Biosystems
BY4741 snf2Δ	BY4741	*snf2::KanMX*	Open Biosystems
BY4741 spt7Δ	BY4741	*spt7::KanMX*	Open Biosystems
BY4741 yta7Δ	BY4741	*yta7::KanMX*	Open Biosystems
ySY101	JKM139	*bdf1::KanMX*	This study
yWJ47	JKM139	*BDF1-3xFLAG-KanMx*	This study
ySY124	JKM139	*RAD52-YFP-KanMX*	This study
yJY115	JKM139	*bdf1::LEU2, RAD52-YFP-KanMX*	This study
yCW007	JKM139	*RFA1-3xFLAG-KANMX*	This study
yCW008	JKM139	*bdf1::LEU2 RFA1-3xFLAG-KanMX*	This study
yCW005	JKM139	*RAD51-3xFLAG-KanMX*	This study
yCW006	JKM139	*bdf1::LEU2 RAD51-3xFLAG-KanMX*	This study
yCW004	JKM139	*RAD53-3xFLAG-KANMX*	This study
yCW003	JKM139	*bdf1::LEU2 RAD53-3xFLAG-KANMX*	This study
yZX067	JKM139	*bdf1-BD1Δ-3xFLAG-KanMX*	This study
yZX068	JKM139	*bdf1-BD2Δ-3xFLAG-KanMX*	This study
yZX069	JKM139	*bdf1-BD1Δ,BD2Δ-3xFLAG-KanMX*	This study
yZX071	JKM139	*bdf1-Y187F-3xFLAG-KanMX*	This study
yZX072	JKM139	*bdf1-Y354F-3xFLAG-KanMX*	This study

Supplemental Table 1 continued**. Yeast strains**

yZX073	JKM139	*bdf1-Y187F,Y354F-3xFLAG-KanMX*	This study
yHY006	tGI354	*bdf1-Y187F,Y354F-3xFLAG-KanMX*	This study
yHY110	BY4732-YAC	*bdf1-Y187F,Y354F-KanMX*	This study
yHZ119	JKM139	*ESA1-3xFLAG-KanMX*	This study
yHY130	JKM139	*NUP49-mCherry-TRP1 + pRS316-BDF1-GFP*	This study
yHY131	JKM139	*NUP49-mCherry-TRP1 + pRS316-bdf1-Y187F,Y354F-GFP*	This study
ySM042	tGI354	*bdf1-RBΔ-3xFLAG-NatMX*	This study
ySM043	tGI354	*bdf1-Y187F,Y354F-RBΔ-3xFLAG-NatMX*	This study
ysm036	JKM139	*bdf1-RBΔ-KanMX*	This study
ysm037	JKM139	*bdf1-Y187F,Y354F-RBΔ-KanMX*	This study
yJY147	JKM139	*BDF1-3xFLAG-3mAID-HphMX, osTIR1-3xHA-TRP1*	This study
yJY157	JKM139	*RAD52-YFP-KanMX BDF1-3xFLAG-3mAID-HphMX, osTIR1-3xHA-TRP1*	This study
yJY158	JKM139	*RAD53-3xFLAG-KanMX BDF1-3xFLAG-3mAID-HphMX, osTIR1-3xHA-TRP1*	This study
yHZ077	JKM139	*bdf1-Y187F,Y354F-NatMX*	This study
yXC780	JKM139	*dot1::KanMX*	This study
yHZ079	JKM139	*dot1::KanMX bdf1-Y187F,Y354F-NatMX*	This study
yHZ032	JKM139	*SGS1-3HA-TRP1 BDF1-3FLAG-KanMX*	This study
yHZ044	JKM139	*SGS1-3HA-TRP1 bdf1-Y187F,Y354F-3FLAG-KanMX*	This study
yHZ016	JKM139	*DNA2-9Myc-TRP1 BDF1-3FLAG-KanMX*	This study
yHZ046	JKM139	*DNA2-9Myc-TRP1 bdf1-Y187F,Y354F-3FLAG-KanMX*	This study
yHZ017	JKM139	*EXO1-9Myc-TRP1 BDF1-3FLAG-KanMX*	This study
yHZ045	JKM139	*EXO1-9Myc-TRP1 bdf1-Y187F,Y354F-3FLAG-KanMX*	This study
yHZ089	tGI354	*bdf1-Y187F,Y354F-NatMX*	This study
yHZ080	tGI354	*dot1::KanMX*	This study
yHZ081	tGI354	*dot1::URA3 bdf1-Y187F,Y354F-NatMX*	This study
yXC671	tGI354	*swr1::NatMX*	This study
yXC631	tGI354	*sgs1::NatMX*	This study
yXC679	tGI354	*fun30::NatMX*	This study
yCW128	JKM139	*RFA1-3xHA-TRP1 bdf1-Y187F,Y354F-3xFLAG-KanMX*	This study
yHZ042	JKM139	*RFA1-3xHA-TRP1 BDF1-3xFLAG-KanMX*	This study
yHZ056	JKM139	*RAD51-3xHA-TRP1*	This study
yHZ057	JKM139	*RAD51-3xHA-TRP1 bdf1-Y187F,Y354F-KanMX*	This study
yXC709	JKM179	*FLAG-HHT1-LEU2*	This study
yHZ069	JKM179	*bdf1-Y187F,Y354F-KanMX FLAG-HHT1-LEU2*	This study
yCW017	JKM139	*MRE11-13xMyc-KanMX*	This study
ySM055	JKM139	*bdf1-Y187F,Y354F-NatMX MRE11-13xMyc-KanMX*	This study
yHZ033	JKM139	*RFA1-3xFLAG-HphMX*	This study
yHZ055	JKM139	*RFA1-3xFLAG-HphMX bdf1-Y187F,Y354F-KanMX*	This study
ySM032	JKM139	*bdf1-Y187F,Y354F-NatMX RAD51-3xFLAG-KanMX*	This study

1. Lee, S. E. et al., Saccharomyces Ku70, mre11/rad50 and RPA proteins regulate adaptation to G2/M arrest after DNA damage.(1998). Cell 94 (3): 399.
2. 2. Ira G. et al., Srs2 and Sgs1-Top3 suppress crossovers during double-strand break repair in yeast. (2003) Cell.115(4):401-11.
3. 3.Wahba L, Amon J D, Koshland D, et al. RNase H and multiple RNA biogenesis factors cooperate to prevent RNA: DNA hybrids from generating genome instability.(2011) Molecular cell 44(6): 978-988.
